# Efficient genome editing using tRNA promoter-driven CRISPR/Cas9 gRNA in *Aspergillus niger*

**DOI:** 10.1371/journal.pone.0202868

**Published:** 2018-08-24

**Authors:** Letian Song, Jean-Paul Ouedraogo, Magdalena Kolbusz, Thi Truc Minh Nguyen, Adrian Tsang

**Affiliations:** Centre for Structural and Functional Genomics, Concordia University, Montreal, Canada; Woosuk University, REPUBLIC OF KOREA

## Abstract

As a powerful tool for fast and precise genome editing, the CRISPR/Cas9 system has been applied in filamentous fungi to improve the efficiency of genome alteration. However, the method of delivering guide RNA (gRNA) remains a bottleneck in performing CRISPR mutagenesis in *Aspergillus* species. Here we report a gRNA transcription driven by endogenous tRNA promoters which include a tRNA gene plus 100 base pairs of upstream sequence. Co-transformation of a *cas9*-expressing plasmid with a linear DNA coding for gRNA demonstrated that 36 of the 37 tRNA promoters tested were able to generate the intended mutation in *A*. *niger*. When gRNA and *cas9* were expressed in a single extra-chromosomal plasmid, the efficiency of gene mutation was as high as 97%. Co-transformation with DNA template for homologous recombination, the CRISPR/Cas9 system resulted ~42% efficiency of gene replacement in a strain with a functioning non-homologous end joining machinery (*kusA*^*+*^), and an efficiency of >90% gene replacement in a *kusA*^*-*^ background. Our results demonstrate that tRNA promoter-mediated gRNA expressions are reliable and efficient in genome editing in *A*. *niger*.

## Introduction

The filamentous fungus *Aspergillus niger* is an important cell factory used in industry for the production of enzymes and organic acids. Owing to its genetic tractability, *A*. *niger* is widely used for research in fungal physiology, biochemistry and biotechnology. The availability of genome sequence of this organism [1,2] has facilitated numerous studies in gene function, gene regulation, primary and secondary metabolism [3,4]. The utility of *A*. *niger* as an industrial cell factory and as a model organism for research can be further improved by the development of a highly efficiency genome-editing system. https://www.ncbi.nlm.nih.gov/pubmed/17259976

Multiple nuclease-based gene targeting methods have been developed to improve the efficiency and precision of generating genetic modifications. These include Zinc-finger nuclease (ZFN) [5], Transcription Activator-Like Effector Nuclease (TALEN) [6], I-*Sce*I meganuclease [7], and Clustered Regularly Interspaced Short Palindromic Repeat / associated nuclease 9 (CRISPR/Cas9) [8]. With the ability of nucleases to make DNA double strand break (DSB) in the host organism, nuclease-based gene targeting methods have been successfully used for gene disruption, knock-in mutation as well as improving heterologous protein production by integrating foreign genes to defined genomic loci [5,9,10]. The type II CRISPR/Cas9 system from *Streptococcus pyogenes* has revolutionized genome editing by providing a simple, flexible and efficient strategy to precisely edit the genomes of mammals [11], plant [12], fly [13], fungi [14] and bacteria [15]. Two components are essential in type II CRISPR/Cas9 system: a functional Cas9 nuclease and a chimeric guide RNA (gRNA) consisted of two regions, a CRISPR RNA (crRNA) harboring 20-nucleotide target-recognizing sequence at the 5’-end and a trans-activating crRNA (tracrRNA) for Cas9 binding. The crRNA is user-defined to match the target genomic locus. It guides the gRNA to form a RNA/DNA hybrid at the target genomic locus and recruits the Cas9 nuclease to generate DNA DSB [16]. Consequently, the CRISPR/Cas9 approach supports the editing of numerous genomic loci.

An efficient promoter that can facilitate gRNA transcription *in vivo* is a bottleneck of adoption of CRISPR/Cas9 system in Aspergilli. So far, in the reported CRISPR/Cas9 platform for *A*. *niger*, RNA polymerase II (Pol II) promoter and terminator were employed to drive the transcription of the gRNA, which required the inclusion of two self-splicing ribozyme sequences flanking the gRNA sequence to free the mature gRNA transcript [14]. The endogenous *U6* promoter, an RNA polymerase III (Pol III) promoter, has been used for CRISPR/Cas9 genome editing in *A*. *oryzae* with an efficiency lower than 20% in generating gene mutations [17]. Another Pol III promoter, *U3* promoter of *A*. *fumigatus* (Af*U3*), was successfully used to transcribe gRNA in *A*. *nidulans*. However, endogenous *U6* and Af*U6* promoters failed in generating functional gRNA in *A*. *nidulans*, which indicates that *U6* promoters have limited function as gRNA promoters in fungi [18,19]. Therefore, it is important to find reliable promoters to drive gRNA transcription *in vivo* that can result in efficient genome editing of *A*. *niger* to expand its utility as a model and industrial organism.

In eukaryote cells, the transfer ribonucleic acid (tRNA) genes are transcribed by Pol III [20]. The transcription of fungal tRNA and other eukaryotes genes is constitutive and is independent of carbon source and cultivation conditions [21,22]. The tRNA promoter includes a region centered approximately 30 base pairs (bp) upstream of the tRNA gene start site and two intragenic sequences, Box A and Box B [20,23,24]. Their short sequences [25] and their self-splicing capacity [26,27] make tRNA promoters suitable for transcribing the short and non-coding gRNA sequence. The self-splicing ability of tRNA has been used to separate multiplexed gRNAs in plants [28] and recently in filamentous fungi [18]. To the best of our knowledge, using tRNA promoter to independently drive gRNA transcription has not been described in filamentous fungi. In this study, we describe the use of tRNA promoters and terminators to drive the gRNA transcription *in vivo* and show that this method is highly efficient in genome editing in *A*. *niger*.

## Results

### The use of tRNA promoters and terminators to drive gRNA transcription in *A*. *niger*

**Fig 1:**
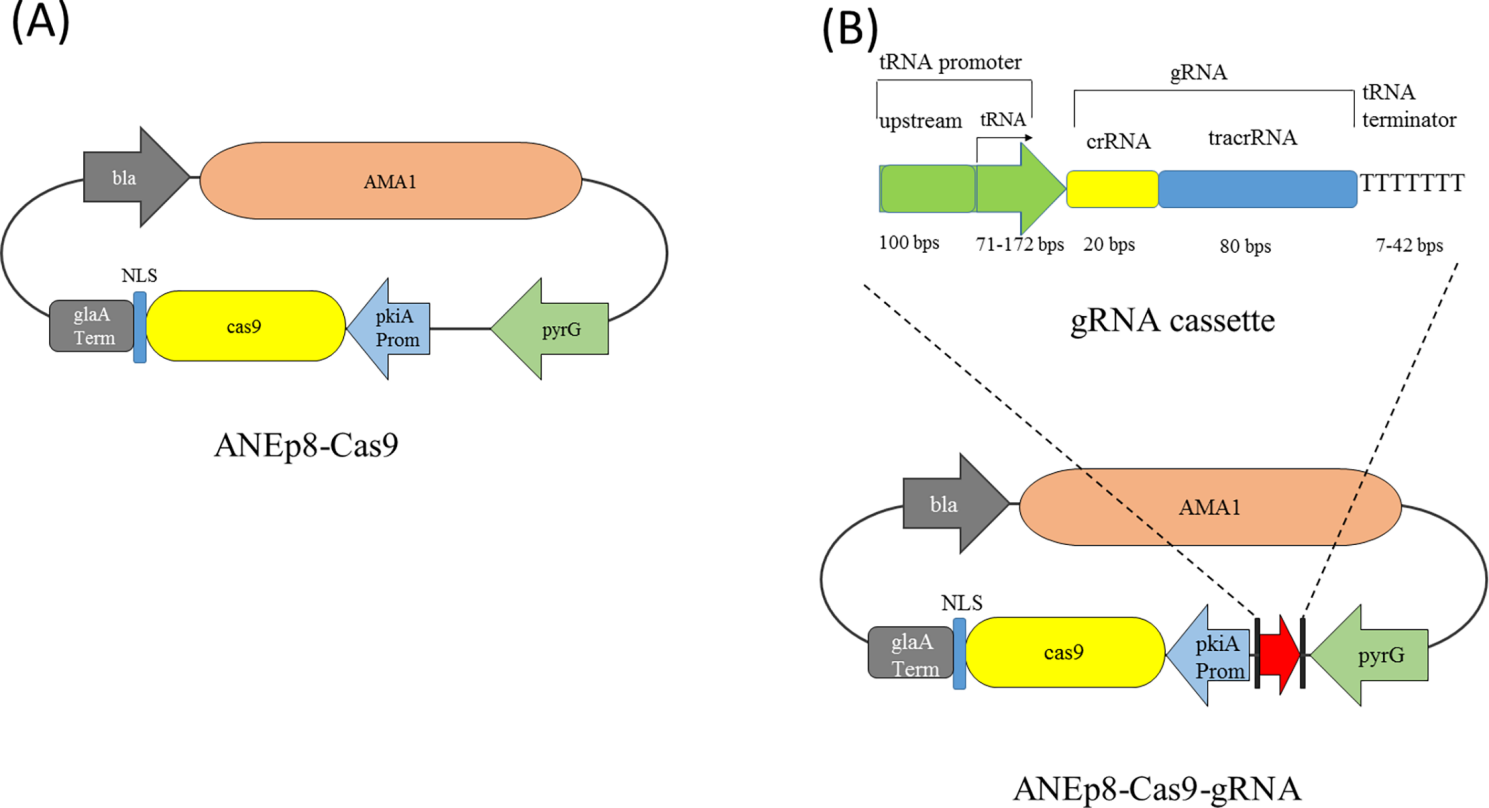
The schematic representation of tRNA-driven CRISPR/Cas9 system adapted for *A*. *niger*. (A) The ANEp8-Cas9 plasmid used to express *cas9* gene. (B) The construct of gRNA cassette and the ANEp8-Cas9-gRNA plasmid bearing both gRNA cassette and *cas9* gene. The tRNA promoter is composed of a tRNA gene and 100 bp upstream sequences. Arrow indicates the start site of transcription. Abbreviations: *bla*, beta-lactamase gene conferring ampicillin resistance; *pkiA* Prom, promoter of the pyruvate kinase gene; *glaA* Term, terminator of the glucoamylase gene; NLS: nuclear localization signal.

We manually curated the genome of the *A*. *niger* NRRL3 (http://gbrowse.fungalgenomics.ca/cgi-bin/gb2/gbrowse/Aspni_nrrl3_public) and identified 284 tRNA genes (chromosomal locations and sequences were listed in S1 Table). Six of the 284 tRNA genes are pseudogenes and three encode selenocystein tRNA. There are 8–24 tRNA genes for 17 of the 20 main amino acids. The three amino acids with fewer than eight corresponding tRNA genes are: tryptophan with one, asparagine with three, and cysteine with four tRNA genes (S1 Table). To determine the ability of tRNA promoters to drive the functional transcription of gRNA, we chose two random tRNA genes from every amino acid except that of asparagine and tryptophan. Three valine tRNAs were chosen due to the lack of intron in one and the difference in intron lengths in the two others. In total, 37 tRNA genes and their flanking sequences to use as promoters and terminators were selected (Table 1). Each gRNA cassette contains the following elements: 1) tRNA promoter which comprises the entire tRNA gene including introns, plus 100 nt upstream sequence; 2) the gRNA sequence that comprises a 20 nt crRNA guiding sequence that targets the *albA* gene (gene ID: NRRL3_00462) and a 80 nt tracrRNA sequence for Cas9 protein binding [29]; and 3) the 7–42 nt of tRNA terminator that spans from the end of tRNA gene to the poly-T stretch. The gene *albA* encodes a polyketide synthase involved in both dihydroxynaphthalene melanin and naphtho-gamma-pyrone synthesis that are required for spore pigmentation [30]. The disruption of *albA* prevents biosynthesis of black pigment, and results in white mutant conidia [14,30]. This phenotypic change provided a visual scoring of mutants directly on the transformation plate. For a quick, initial test of the ability of tRNA promoters and terminators to regulate gRNA transcription, we co-transformed the 37 linear gRNA cassettes independently into *A*. *niger* with *cas9*-bearing plasmid ANEp8-Cas9 (Fig 1A). The ANEp8-Cas9 plasmid was derived from the plasmid ANEp8 [31] that contains AMA1 sequence allowing extra-chromosomal replication [32] and the *pyrG* selection marker.

**Table 1:** Functional testing of 37 tRNA promoters and terminators in CRISPR/cas9 genome editing.

tRNA promoter	Length of tRNA gene (bp)	Length of intron (bp)	Anticodon	Length of tRNA promoter (bp)	Length of tRNA terminator (bp)	Number of total colonies in transformation plates*	Number of white colonies (*albA* mutant)
tRNA^Ala5^	72	0	AGC	172	16	88	9
tRNA^Ala23^	72	0	AGC	172	7	36	2
tRNA^Arg8^	72	0	ACG	172	9	96	12
tRNA^Arg21^	72	0	ACG	172	9	71	8
tRNA^Asp2^	91	19	GTC	191	18	124	12
tRNA^Asp5^	90	18	GTC	190	11	65	2
tRNA^Cys1^	72	0	GCA	172	11	100	7
tRNA^Cys2^	72	0	GCA	172	15	72	5
tRNA^Gln1^	72	0	CTG	172	16	72	6
tRNA^Gln2^	92	20	TTG	192	20	102	6
tRNA^Glu1^	72	0	TTC	172	17	56	4
tRNA^Glu12^	71	0	TTC	171	16	63	3
tRNA^Gly5^	71	0	GCC	171	26	68	0
tRNA^Gly13^	71	0	GCC	171	16	34	3
tRNA^His2^	84	12	GTG	184	42	62	4
tRNA^His3^	71	0	GTG	171	32	82	4
tRNA^Ile4^	74	0	AAT	174	16	60	3
tRNA^Ile8^	74	0	AAT	174	17	94	4
tRNA^Leu6^	111	27	AAG	211	17	148	8
tRNA^Leu14^	112	27	AAG	212	13	46	1
tRNA^Lys6^	73	0	CTT	173	16	124	6
tRNA^Lys17^	73	0	CTT	173	18	82	5
tRNA^Met4^	72	0	CAT	172	15	84	2
tRNA^Met9^	72	0	CAT	172	21	78	3
tRNA^Phe2^	73	0	GAA	173	17	60	2
tRNA^Phe12^	73	0	GAA	173	11	88	10
tRNA^Pro1^	91	19	AGG	191	17	57	7
tRNA^Pro3^	91	19	AGG	191	17	68	3
tRNA^Ser4^	105	23	GCT	205	13	98	4
tRNA^Ser7^	81	0	AGA	181	15	90	4
tRNA^Thr5^	73	0	AGT	173	15	46	4
tRNA^Thr6^	73	0	AGT	173	13	48	4
tRNA^Tyr3^	88	13	GTA	188	22	63	6
tRNA^Ty7^	88	13	GTA	188	21	42	2
tRNA^Val1^	74	0	CAC	174	18	33	4
tRNA^Val4^	96	23	CAC	196	25	45	1
tRNA^Val15^	87	14	TAC	187	18	64	7

* The colonies number was from single transformations

In the control plate where the cells were transformed with ANEp8-Cas9 alone, only black colonies appeared (S1A Fig). Except in the case of tRNA^Gly5^, all co-transformation yielded a mixture of black and white mutant colonies. These results suggest that almost all endogenous tRNA promoters and terminators are able to transcribe functional gRNA in the CRISPR/Cas9 system in *A*. *niger*. However, the co-transformation method resulted low inactivation rate of *albA* gene, ranging between 2 to 13% (Table 1). The relatively low rates of mutation using this co-transformation method is expected because the frequency of maintaining extra-chromosomal plasmid harboring the selectable marker is much higher than that of maintaining the integrated exogenous DNA [32]. In other words, only an uncontrolled subpopulation of transformants in these experiments is predicted to contain the linear gRNA cassette. Consequently, this initial experiment only reveals that most endogenous tRNA promoters and terminators are able to drive gRNA transcription and support CRISPR/Cas9 mutagenesis in *A*. *niger*, it does not provide quantitative information on the efficiency of different tRNA promoters in generating functional gRNA.

### tRNA promoters are highly efficient in driving gRNA transcription

To compare the efficiency of different tRNA promoters in CRISPR/Cas9 system, the gRNA cassette was cloned into ANEp8-Cas9 to generate plasmid ANEp8-Cas9-gRNA (Fig 1B). We used extra-chromosomal plasmid for three reasons: 1) it supports high frequency of transformation [33]; 2) the transcription level of *cas9* and gRNA are expected to be similar among transformants as they are not influenced by the site of integration; and 3) the plasmid can be removed easily by counter selection, leaving no exogenous DNA.

From the 36 positive tRNA-driven gRNAs (targeting *albA*) tested in the above co-transformation experiment, we chose eleven gRNA cassettes to construct plasmids containing both *cas9* and gRNA. The eleven cassettes include tRNAs carrying hydrophilic (tRNA^Arg21^, tRNA^Cys1^ and tRNA^Gln2^), polar aliphatic (tRNA^Asp5^), aliphatic hydrophobic (tRNA^Ala5^, tRNA^Leu6^, tRNA^Pro1^ tRNA^Val1; 4; 15^) and aromatic hydrophobic (tRNA^Tyr3^) amino acids. Among them three valine tRNA promoters were included because their tRNA genes contain either no intron or different intron lengths (Table 1).

The eleven ANEp8-Cas9-gRNA plasmids were transformed individually into *A*. *niger* strain N593 (Table 2). The results showed that ten out of eleven cassettes gave rise to transformants of which 82–96% were mutants (Table 3 and S1A Fig). The tRNA^Gln2^ cassette resulted in significantly lower mutation rate of 15±6%. Additionally, the three tRNA^Val^ promoters displayed a similarly high level of mutational efficiency, suggesting that tRNA introns do not influence its promoter capability in gRNA transcription.

**Table 2:** Strains and plasmids used in this study.

***A*. *niger* strains**	**Genotype and/or Description**	**References**
NRRL3	Wild type	
N593 (ATCC 64973)	*cspA pyrA6*	[34]
NRRL2270 (ATCC11414)	Citric acid producer	[35]
NRRL2270*ΔpyrG*	Uridine auxotroph	This study
NRRL2270*ΔpyrGΔkusA*	Uridine auxotroph and NHEJ deficient	This study
**Plasmids**		
ANEp8-Cas9	Extra-chromosomal *cas9* expressing plasmid	This study
ANEp8-Cas9-gRNAalbA	Extra-chromosomal *cas9* expressing plasmid with gRNA targeting *albA* locus	This study
ANEp8-Cas9-gRNAolvA	Extra-chromosomal *cas9* expressing plasmid with gRNA targeting *olvA* locus	This study
ANEp8-Cas9-gRNAglaA	Extra-chromosomal *cas9* expressing plasmid with gRNA targeting *glaA* locus	This study

**Table 3:** Gene disruption rate mediated by using eleven different tRNA promoters in ANEp8-Cas9-gRNA plasmid.

tRNA promoter	Gene disruption rate (%)*	
	*ΔalbA*	*ΔolvA*
tRNA^Ala5^	93±2	nd^†^
tRNA^Arg21^	92±2	97±1
tRNA^Asp5^	96±1	nd
tRNA^Cys1^	82±6	96±2
tRNA^Gln2^	15±6	13±2
tRNA^Leu6^	82±7	95±2
tRNA^Pro1^	93±1	88±2
tRNA^Tyr3^	82±11	nd
tRNA^Val15^	82±14	nd
tRNA^Val4^	90±3	nd
tRNA^Val1^	94±1	nd

^*^The rate was calculated as the percentage of mutant colonies in all transformants. Results were based on triplicate transformations.

^†^nd: not determined.

To test the reliability of tRNA promoters in driving functional gRNA transcription in the CRISPR/Cas9 system, we targeted another gene *olvA* (ID: NRRL3_01039) that is also involved in the conidial pigmentation pathway. The loss of OlvA function results in olive-colored conidial mutants [30] (S1 Fig). We used five different tRNA promoters to drive gRNA to target *olvA*, four of which were randomly selected from the ten promoters that yielded >80% mutational frequency. We included the tRNA^Gln2^ promoter to assess whether the low frequency of obtaining mutants is promoter dependent. The mutation rate of *olvA* was very similar to that observed towards *albA* (Table 3). A mutation rate of 13±2% was achieved by using tRNA^Gln2^, while tRNA^Arg21^, tRNA^Cys1^, tRNA^Leu6^, and tRNA^Pro1^ were able to induce mutation rates of between 88 to 97%. These results indicate that 1) the efficiency of gene editing by the CRISPR/Cas9 system is promoter-dependent; and 2) most endogenous tRNAs are very efficient and reliable in driving gRNA transcription in the CRISPR/Cas9 system.

### Both deletions and insertions are generated by tRNA promoter-driven CRISPR/Cas9 mutagenesis

To determine the nature of CRISPR/Cas9-mediated mutations, we PCR-amplified the target genomic regions of the phenotypic mutants and sequenced the PCR products. Thirty transformants were analyzed, representing 15 *albA* and 15 *olvA* mutants mediated by five different tRNA promoters. These fragments covered the regions of *albA* and *olvA* that were expected to be cleaved by Cas9. Table 4 shows that deletions were the predominant mutation with 22 out of 30 of Cas9-induced mutations appeared as short deletions in the range of 1 to 27 bp at the Cas9 cleavage site. Additionally, five mutants contained long deletions of 612–1051 bp. Insertions were observed in three mutants. Sequence analysis indicated that the inserted sequences were derived from ANEp8-Cas9-gRNA plasmid. These observations suggest that double strand break mediated by Cas9 induces error-prone repair that resulted in mutation of the targeted gene in *A*. *niger*. The results also suggest that the extra-chromosomal plasmid can function as pseudo-donor template in the insertion of sequences.

**Table 4:** Mutations generated by tRNA promoter-driven CRISPR/Cas9 mutagenesis.

tRNA promoter	Target Gene	Mutant number	Deletion*	Insertion	Mismatch
tRNA^Arg21^	*albA*	1	6 bp (+1 - +6)		
	*albA*	2	6 bp (+1 - +6)		
	*albA*	3	4 bp (+1 - +4)		
	*olvA*	1	8 bp (+1 - +8)		
	*olvA*	2	612 bp (-11 - +429; +438 - + 609)		
	*olvA*	3		72 bp (-1 - +1)	
tRNA^Cys1^	*albA*	1	27 bp (-27 - -1)		
	*albA*	2	854 bp (-854 - -1)		
	*albA*	3	815 bp (-813 - +2)		1 bp (T-817C)
	*olvA*	1	18 bp (+1 - +18)		
	*olvA*	2	10 bp (-2 - +8)		
	*olvA*	3	7 bp (-1 - +6)		
tRNA^Gln2^	*albA*	1		39 bp (-2 - -1)	
	*albA*	2	12 bp (-1 - +11)		
	*albA*	3	22 bp (-22 - -1)		
	*olvA*	1	13 bp (-12 - +1)		
	*olvA*	2	1096 bp (-1038 - +58)		
	*olvA*	3	11 bp (-8 - +3)		
tRNA^Leu6^	*albA*	1	1 bp (-1)		
	*albA*	2	15 bp (-3 - +12)		
	*albA*	3	979 bp (-979 - -1)		
	*olvA*	1	1 bp (-1)		
	*olvA*	2		88 bp (-1 - +4)	
	*olvA*	3	13 bp (-13 - -1)		
tRNA^Pro1^	*albA*	1	6 bp (-7 - -2)		
	*albA*	2	4 bp (-3 - +1)		1 bp (A-6G)
	*albA*	3	2 bp (-2 - -1)		
	*olvA*	1	1 bp (-1)		
	*olvA*	2	2 bp (-2 - -1)		
	*olvA*	3	1 bp (-1)		

^*^The mutation locations are given in brackets. The Cas9 cleavage site is defined as 3 bp upstream of the PAM sequence and is designated position "0". The negative and positive coordinates indicate mutations locate at 5’ and 3’ to the position 0, respectively.

### CRISPR/Cas9 mediates gene replacement in *A*. *niger kusA*^*+*^ and *kusA*^*-*^ backgrounds with different efficiencies

To evaluate how CRISPR/Cas9-mediated DSB can improve gene targeted replacement in *A*. *niger*, we constructed a ANEp8-Cas9-gRNAglaA plasmid targeting the *glaA* locus, which is a highly expressed gene encoding glucoamylase, and a linear gene replacement cassette as donor template (S2 Fig). The linear donor template comprised the coding region of *adaR* (gene ID: NRRL3_09545) flanked by 600 bp of *glaA* promoter and terminator sequences. The *adaR* gene encodes a transcription regulator involved in the biosynthesis of TAN-1612/BMS-192548, a pigmented polyketide secondary metabolite [36]. The integration and overexpression of *adaR* yields orange colonies because of the overproduction of TAN-1612/BMS-192548 [36]. Orange colonies can be directly counted on transformation plates. As a control, we transformed donor DNA with the plasmid ANEp8-Cas9 without the gRNA cassette. In addition, we created the *kusA*^*-*^ mutant strain according to Meyer *et al*. [35] and described in S3 and S4 Figs to evaluate the role of non-homologous end joining (NHEJ) DNA repair in mediating CRISPR/Cas9 mutagenesis. Mutants deficient in KusA are defective in NHEJ and cannot repair DNA DSBs [37,38]. Previous studies showed improved homologous recombination in NHEJ-deficient *A*. *niger* strains [38–40]. However, little is known about the efficiency of homologous recombination when DSB is mediated by CRISPR/Cas9.

In order to easily distinguish gene integration mutants from abortive or young transformants, nine orange colonies from each transformation experiment were chosen for PCR screening using primers outside the *glaA* locus (S2 Fig). Table 5 shows that in the *kusA*^+^ background, without the gRNA no gene replacement occurred. In the presence of the gRNA in the *kusA*^+^ background, about 75% of the transformants displayed orange colonies and 5 of 9 orange colonies tested were the results of homologous recombination, suggesting a frequency of gene replacement of about 42%. In the *kusA*^*-*^ background, the absence of gRNA yielded 21 orange colonies out of 75 transformants with 6 of 9 orange colonies tested positive for homologous recombination, suggesting a frequency of gene replacement of about 20%. This finding supports previous observation that the turning off of NHEJ repair that improves homologous recombination in *A*. *niger* [39,40]. In the *kusA*^*-*^ background and in the presence of gRNA, orange colonies comprised about 93% of the transformants with all 9 orange colonies tested positive for homologous recombination. This would imply a gene replacement rate of ~93%. In addition, in the *kusA*^*-*^ strain, no transformant was found in the experiment in which only the ANEp8-Cas9-gRNAglaA plasmid was transformed because fungal cells could not repair the DSB in the absence of a repair donor template (Table 5). Based on this reasoning, we would expect 100%, instead of 93%, gene replacement frequency in the situation where we have functional gRNA and a gene replacement DNA template in the *kusA*^*-*^ background. The few non-orange transformants could be the result of less than 100% efficiency of the gRNA and/or some of the young transformants scored did not have sufficient time to manifest the mutant phenotype. Since *kusA*^*-*^ strains cannot repair DSB, constructing mutants in the *kusA*^*-*^ genetic background using CRISPR/Cas9 and rescue templates has the advantage that off-target mutations would be minimized as ectopic mutations would result in unrepaired chromosomal breaks and cell lethality.

**Table 5:** CRISPR/Cas9-mediated targeting homologous integration in *A*. *niger*.

CRISPR plasmid	*adaR* donor template	NHEJ competent strain (*kusA*^*+*^)			NHEJ deficient strain (*kusA*^*-*^)		
		Total colonies*	Orange colonies*^†^	Gene replacement mutants / Screened mutants^‡^	Total colonies*	Orange colonies*^†^	Gene replacement mutants / Screened mutants^‡^
ANEp8-Cas9	Yes	125 ± 3	15 ± 1	0/9	75 ± 1	21 ± 5	6/9
ANEp8-Cas9-gRNAglaA	Yes	106 ± 4	80 ± 3	5/9	88 ± 7	82 ± 6	9/9
ANEp8-Cas9-gRNAglaA	No	98 ± 6	-	-	0	-	-

^*^The value was calculated from duplicate independent transformations and the average is mentioned.

^**†**^The mutant number was counted according to the phenotype of orange color in the primary transformants.

^**‡**^From each scenario, nine of the orange colored transformants were selected to screen for gene replacement by PCR.

## Discussion

Besides an active Cas9 protein, a functional gRNA is an essential component in CRISPR/Cas9 system to engineer the host genome. For delivering gRNA *in vivo*, the formation of functional gRNA requires an efficient promoter to drive gRNA transcription and a splicing mechanism to form mature gRNA transcript. In this study, we describe a strategy using Pol III tRNA promoters to drive gRNA transcription. This study shows that the self-splicing ability of tRNAs is highly efficient in generating functional gRNAs.

Since 2015, the CRISPR/Cas9 system in genome editing has been established in several genera of filamentous fungi. As summarized in Table 6, the promoter selection has shifted from Pol II promoter (e.g. *pgdA* and *trpC* promoters) to Pol III promoters for the *in vivo* transcription of gRNA. This is because Pol III promoter is more suitable for *in vivo* transcription of short non-coding transcripts such as gRNA [20,41]. In addition, the short length of Pol III promoters and terminators makes it easier to construct gRNA cassette. Among Pol III promoters used in filamentous fungi CRISPR/Cas9 platforms, the species-specific *U*6 promoter is the most widely used [17,42–47], and the *SNR*52 promoter from *Saccharomyces cerevisiae* has been tested in *Neurospora crassa* [48] and *A*. *fumigatus* [49] (Table 6). In this study, the tRNA promoters contain the entire sequence of tRNA gene plus 100 nt upstream sequence with a total sequence varying from 171 to 272 nt (S2 Table), which is similar as the lengths of *U6* (200 to 250 nt) and *S*. *cerevisiae SNR52* (269 nt) promoters [29,50]. The main challenge of current Pol III gRNA promoters such as U3 and U6 is that the same type of promoter results in highly variable gene editing efficiency amongst different species. Unlike *U6* promoter that is an upstream element outside of the transcription start site [50], the tRNA gene is transcribed together with gRNA sequence [20,51]. In the cell the tRNA transcript is posttranscriptionally cleaved at the 3‘-end by RNase Z [27,28]. Accordingly, tRNA promoter regulated system is able to release mature functional gRNA transcript as demonstrated in this study and elsewhere [52].

**Table 6:** Summary of CRISPR/Cas9-mediated gene mutation and replacement efficiencies in filamentous fungi.

Organism	gRNA cassette components			Gene replacement rate (%)	Gene disruption rate (%)	Expression plasmid/other method	References
	Promoter	Ribozyme splicing sequences	Terminator				
*A*. *niger*	tRNA	No	tRNA	42% or 93%^*^	13–15% or 82–97%^**†**^	Extrachromosomal (AMA1)	This study
*A*. *nidulans*	*gpdA*	Yes	*trpC*	90%	20–30%	Extrachromosomal (AMA1)	[14]
*A*. *aculeatus*	*gpdA*	Yes	*trpC*	65%	> 70%	Extrachromosomal (AMA1)	[14]
*A*. *fumigatus*	*snr52*	No	*sup4*	nd^**‡**^	25–53%	Integrative	[49]
*A*. *fumigatus*	*U6-1/2/3*	No	*U6*	63%	43%	Extrachromosomal (AMA1)	[43]
*A*. *oryzae*	*U6*	No	*U6*	nd	10–20%	Integrative	[17]
*N*. *crassa*	*snr52*	No	*sup4*	nd	nd	Integrative	[48]
*Pyricularia oryzae*	*U6-1/2* t*rpC*	No Yes	*U6* *trpC*	36–84% 10–27%	nd nd	Integrative	[42]
*T*. *reesei*	*In vitro* transcription			93–100%	nd	*in vitro*	[53]
*Ustilago maydis*	*U6*	No	*U6*	nd	70–100%	Extrachromosomal (ARS)	[44]
*Penicillium chrysogenum*	*U6* tRNA	No No	*U6* tRNA	100% nd	nd nd	Extrachromosomal (AMA1)	[45]
*A*. *carbonarius*	*gpdA*	Yes	*trpC*	100%	nd	Extrachromosomal (AMA1)	[54]
*Coprinopsis cinerea*	*U6*	No	*U6*	nd	21%	Integrative	[47]
*Myceliophthora thermophila*	*U6*	No	*U6*	95%	nd	Integrative	[46]
*M*. *heterothallica*	*U6*	No	*U6*	90%	nd	Integrative	[46]

^*^The value of 42% and 93% were obtained from experiments in *A*. *niger kusA*^*-*^ and *kusA*^*+*^ strains respectively using tRNA^Pro1^ as gRNA cassette promoter.

^**†**^The 13–15% correspond to tRNA^Gln2^ promoter, and the 82–97% correspond to the other ten tRNA promoters as described in this study.

^**‡**^nd: not determined.

By introducing upstream sequences, the tRNA promoters in our constructs include all external and internal binding sites of Pol III transcription factors needed for transcription to proceed normally. Except tRNA^Gln2^, the tRNA promoters tested in this study lead to high rate of gene mutation (82–97%). In endogenous *U6* promoter-driven platforms, the mutation rate is highly variable among different fungal species and ranges from 10 to 100% (Table 6). In a recent study, the endogenous *U6* promoter and the heterologous *AfU6* promoter failed to transcribe gRNA in CRISPR/Cas9 system in *A*. *nidulans* [18]. In addition, gRNAs transcribed by *S*. *cerevisiae SNR52* promoter resulted in mutation rates of between 25 to 53% in *A*. *fumigatus* [49] (Table 6). These studies show that the type of promoters used to transcribe gRNA has a high impact in CRISPR/Cas9 activity. Overall results show that expressing Cas9 and the gRNA in single plasmid improve gene deletion and gene replacement. In addition no difference on growth was observed when transformed *A*. *niger* with ANEp8-Cas9 or ANEp8-Cas9-gRNA in the present study, except the color phenotype (S1A and S1B Fig). This study provides a broad list of highly efficient tRNA promoters for gRNA transcription.

In *A*. *niger* and other eukaryotes, DSB created in the genome is repaired by NHEJ or by homologous recombination [55]. The NHEJ is the most dominant pathway in *A*. *niger* and the frequency of homologous recombination increases with the deletion of the *kusA* gene, which is involved in the NHEJ repair mechanism [56]. In this study, we tested CRISPR/Cas9 mediated gene replacement by homologous recombination in both *kusA*^*-*^ and *kusA*^*+*^ strains. By targeting the *glaA* locus, we demonstrated that the CRISPR/Cas9 method can increase the frequency of gene replacement in *A*. *niger* (Table 5). In gene replacement mediated by CRISPR/Cas9 activity, our results are comparable to the results obtained in filamentous fungi such as *A*. *nidulans*, *Trichoderma reesei* and *Myceliophthora thermophila*, and show higher frequency comparing to findings in *A*. *aculeatus*, *A*. *fumigatus*, *Penicillium chrysogenum* and *A*. *carbonarius* (Table 6). In summary, the tRNA promoter-driven gRNA transcription system is very efficient to generate DNA double-strand break for gene mutation mediated by NHEJ and for gene replacement by homologous recombination in *kusA*^*+*^ and *kusA*^*-*^ strains. This powerful tool will facilitate further improvement of the industrial fungus *A*. *niger*.

## Material and methods

### CRISPR design

#### tRNA gene prediction and selection

The tRNA gene and promoter sequences for this study were retrieved from the genome sequence of *A*. *niger* NRRL3 (data not published, available at http://genome.fungalgenomics.ca). We used four tRNA gene electronic tools tRNAscan-SE version 1.3 [57], SPLITSX [58], ARAGORN [59] and tRNAfinder [60] to predict all potential cytoplasmic tRNA genes in the nuclear genome of *A*. *niger* NRRL3. A total of 451 tRNA gene models were predicted by the four tools. We manually reviewed the predicted models to ensure that they conform to the length and structure of tRNAs and that the terminator contains a stretch of consecutive thymidines (poly-T) [61,62]. Where multiple non-identical models are detected in the same genomic region, we chose the consensus model predicted by more than one program. Since no fungal tRNA gene has been found to have intron exceeding 100 nucleotides [63], we removed 14 tRNA models predicted by ARAGORN to have introns >100 nucleotides. The final set contains 284 tRNA gene models (S1 Table).

#### Identification of guide sequences

We used Geneious R9.1 software [64] to search 20 bp guide sequences against the *A*. *niger* NRRL3 genome. S3 Table shows the guide sequences used in this study, and none of them possesses off-target potential.

#### Construction of gRNA cassette

The gRNA cassettes were assembled by fusion PCR of two DNA fragments, and the primers used were listed in S4 Table. The first fragment was the tRNA promoter that was amplified from *A*. *niger* genomic DNA and the 20 bp guide sequence was added at the 3’-end through PCR using as overlapping sequence. The second fragment was amplified from the synthesized 80 bp tracRNA template [29] with the 20 bp guide and tRNA terminator sequences (S3 Table) added at 5‘and 3‘ends respectively via PCR. In the fusion PCR, 0.5μL of each unpurified PCR fragments were used as template in a 100 μL reaction and performed with Phusion DNA polymerase (Thermo Scientific).

### Plasmid construction

*Escherichia coli* DH5ɑ was used as the host strain for maintenance and production of plasmid DNA.

#### Construction of plasmid ANEp8-Cas9

The coding sequence of *S*. *pyogenes cas9* (Uniprot Q99ZW2) was codon optimized for translation in *A*. *niger*. A nucleotide sequence encoding a nuclear localization signal (NLS) peptide SRADPKKKRKV was fused to the carboxy terminus of *cas9*. The DNA sequence of Cas9-NLS was synthesized by IDT Inc. (IA, USA), and amplified by primers Cas9-F and Cas9-R (S4 and S5 Tables) to add *Pac*I and *Fse*I restriction sites at the 5’ and 3’ ends, respectively. Restriction enzyme digests by *Pac*I and *Fse*I were used to clone *cas9-NLS* downstream of the *pkiA* promoter in the expression vector ANEp8 [31]. The resulting *cas9*-containing plasmid was designated as ANEp8-Cas9 (S5 Table).

#### Construction of plasmid ANEp8-Cas9-gRNA

A 38-bp ligation independent cloning (LIC) site centered with *Swa*I restriction site (S5 Fig) was introduced into ANEp8-Cas9 plasmid via PCR amplification. The resulting plasmid was used as host vector to harbor the gRNA cassette by using the LIC method [65]. The gRNA cassette used for plasmid construction was amplified with a pair of end primers to link with LIC sequence sites at the both sides as illustrated in S5 Fig, to generate complementary single-strand overhangs between ANEp8-Cas9 vector and gRNA cassette insert. The linearized ANEp8-Cas9 and the gRNA cassette DNA (ending with LIC tails) were treated by T4 DNA polymerase in the presence of dGTP and dCTP, respectively. The 20 μL reaction mixture contained 0.2 pmol of DNA, 0.8 μL of 100 mM dithiothreitol, 2 μL of 25 mM dGTP or dCTP, and 3 U of T4 DNA polymerase in NEB buffer 2.1. The reaction was carried out at 22°C for 30 minutes followed by enzyme inactivation by heating at 75°C for 20 minutes. The insert and vector were mixed in a 3:1 molar ratio. To achieve annealing, the mixture was first heated at 60°C for 5 minutes and then gradually decreased to 4°C (reduce 0.1°C per second). The annealed products were transformed into *E*.*coli* DH5α competent cells to generate plasmid ANEp8-Cas9-gRNA. All plasmids are available upon request.

### Construction of *adaR* cassette for gene replacement at the *glaA* locus

The linear *adaR* (gene ID: NRRL3_09545) cassette with *glaA* flanking regions was constructed as shown in S2 Fig and S5 Table. Using genomic DNA of the *A*. *niger* strain NRRL2270 as template, 600 bp of 5’and 3’ regions flanking the coding region of the *glaA* gene as well as the *adaR* gene were amplified by primers with complementary ends (S4 Table). Based on their terminal overlaps, the three fragments were joined through fusion PCR amplification, resulting in an *adaR* cassette that was used for gene replacement at the *glaA* locus. A total of 5 μg of purified linear *adaR* cassettes were used in transformation. The PCR screening for targeted integration was carried out by using primers Fw_glaA-ext and Rv_glaA-ext (S2 Fig and S4 Table).

### CRISPR/Cas9 genome editing in *A*. *niger*

#### Strain, media and growth conditions

Table 2 lists the *A*. *niger* strains used in this study. The uridine auxotrophic *A*. *niger* strain N593 was used for gene editing and to test tRNA promoter efficiency by CRISPR/Cas9. The strains NRRL2270Δ*pyrG* and NRRL2270Δ*pyrG*Δ*kusA* were used for testing gene replacement by CRISPR/Cas9. All *A*. *niger* strains were grown on minimal medium [66] or on complete medium (minimal medium supplemented with 5 g/L of yeast extract and 1 g/L of casamino acids). As required, a final concentration of 1.3 mg/mL of 5-fluoroorotic acid (5-FOA) and/or 10 mM of uridine were added. The liquid media were supplemented with 15% D-maltose. The transformants were cultured at 30°C for 4 days.

#### Transformation, mutant purification and screening

Transformation and genomic DNA isolation of *A*. *niger* strains were performed as described previously [3]. All transformations were carried out using 1 μg of plasmid DNA together with 5 μg of linear cassette DNA when needed. Triplicate transformations were performed for each tRNA promoter tested, and duplicate transformations were carried out for the gene replacement of *glaA*. Mutant clones were visually identified according to conidia color. The CRISPR efficiency was calculated by dividing the number of mutant colonies by the total number of transformants. Three mutant colonies were randomly selected from the transformation plate and streak-purified twice on new minimal medium plates without uridine. Mutation patterns were analyzed by PCR amplification of *albA* (1.8 kb) and *olvA* (2.2 kb) sequences surrounding the CRISPR/Cas9 cut site using primers listed in S4 Table. Amplified DNA bands were visualized on agarose gel. Detailed information on the mutations was determined by DNA sequencing of the PCR products.

## Supporting information

S1 Fig An example of transformation plates for gene mutations and growth of purified transformants. A) From left to right are the transformation plates of *A*. *niger* N593 cells transformed with ANEp8-Cas9 plasmid (only expressed *cas9*), and ANEp8-Cas9-gRNA plasmid bearing tRNA^Pro1^-driven gRNA to disrupt *albA* (98% efficiency) and *olvA* (95% efficiency) respectively. B) Growth phenotype of purified colonies of *A*. *niger* transformed with ANEp8-Cas9 and ANEp8-Cas9-gRNAolvA.
(PDF)

S2 Fig Gene replacement of *glaA* gene. (A) Schematic illustration of targeted replacement by homologous recombination. (B) PCR screening for CRISPR/Cas9-mediated homologous integration among the nine-selected orange transformants from *kusA*^*+*^ (left) and *kusA*^*-*^ (right) strains. The PCR was performed using their genomic DNA as template with primers outside of *glaA* locus. Replacement of *glaA* by *adaR* gave a 4.2 kb PCR fragment. Random integration of *adaR* cassette would leave the *glaA* locus intact, resulting in a 3.8 kb PCR fragment.
(PDF)

S3 Fig Construction of *kusA* deletion cassette and strategy to generate NRRL2270*ΔkusAΔpyrG* strain. (A) Fusions PCR to construct the *kusA* deletion cassette by including 500 bps repeat of the 3’ UTR in 5’ of the pyrG selection marker to facilitate the loop out of the selection marker. (B) Loop out strategy of the pyrG selection marker to generate ***Δ****kusA****Δ****pyrG* strain for subsequent transformation with the same selection marker: (1) Homologous integration of the *kusA* deletion cassette to remove the *kusA* gene in *A*. *niger*; (2) Generation of ***Δ****kusA****Δ****pyrG* genotype in presence of 5-FOA plus uridine to facilitate *pyrG* loop out (3).
(PDF)

S4 Fig PCR screening of transformants of *ΔkusApyrG*^*+*^ and *ΔkusAΔpyrG* genotypes respectively and the growth comparison with parental strain. (A) Screening of transformants for ***Δ****kusApyrG*^*+*^ genotype with primers binding externally to the *kusA* locus and primers binding within the *pyrG* selection marker. Two PCR bands with size of 3 kb and 2.5 kb respectively are expected by using primer pairs P1_ext/P6_int and P5_int/P8_ext. Transformants 3, 6, 9 and 13 have the deletion cassette integrated in the *kusA* locus and have ***Δ****kusApyrG*^*+*^ genotype, when transformants 4, 5, 7, 8, 10, 11, 12 and 14 have partial integration of the deletion cassette. For transformants lacking *pyrG*, the primers are not able to bind and amplify the gDNA of the parental strain (PS) NRRL2270*pyrG*^*-*^. (B) Screening for transformants with *pyrG* loop out. Spores of transformant 3 (NRRL2270***Δ****kusApyrG*^*+*^) have been plated on minimal medium containing 5-FOA and uridine for 5 days. Colonies growing on 5-FOA are selected for screening *pyrG* selection marker loop out phenotype by PCR. A correct loop out of the *pyrG* marker is characterized by a PCR giving a band size of 2.2 kb, while a strain failed in *pyrG* loop out will display a band of 4 kb. Transformant 3’ with the correct loop out of the *pyrG* selection marker is NRRL2270***Δ****kusA****Δ****pyrG* and has been selected for further work. (C) Growth comparison of NRRL2270***Δ****pyrG* and NRRL2270***Δ****kusA****Δ****pyrG*. One million spores of strains NRRL2270***Δ****kusA****Δ****pyrG* and NRRL2270***Δ****pyrG* were plated on the uridine-contained minimal or complete medium, and incubated at 30°C for 3 days.
(PDF)

S5 Fig The construction of ANEp8-Cas9-gRNA plasmid using LIC method. In ANEp8-Cas9 linearized vector, the single-strand 5' overhangs for LIC cloning were achieved by 3' →5' exonuclease activity of T4 DNA polymerase in the presence of dGTP. For gRNA cassette, the LIC tails were added to the ends via PCR, and the reverse complementing overhangs were generated by the same T4 DNA polymerase treatment process with dCTP. Through base pairing, plasmid ANEp8-Cas9-gRNA was assembled by annealing the complementary sequences between gRNA insert and plasmid vector. The complementary LIC sequences in the gRNA insert and ANEp8-Cas9 vector are shown in the figure.
(PDF)

S1 Table The 284 predicted tRNA genes from *A*. *niger* NRRL3 genome. (XLSX)

S2 Table DNA sequence of the 37 tRNA promoters and terminators used in the functional test. (XLSX)

S3 Table The guide sequences used in this study. (DOCX)

S4 Table Primers used in this study. (XLSX)

S5 Table DNA sequences of plasmids ANEp8-Cas9 and ANEp8-Cas9-gRNAglaA, and linear construct of *adaR*. (PDF)
