# Supplementary figures and images for: Efficient genome editing using tRNA promoter-driven CRISPR/Cas9 gRNA in Aspergillus niger
Source: PLoS One. 2018 Aug 24;13(8):e0202868. doi: 10.1371/journal.pone.0202868 (PMC6108506; doi:10.1371/journal.pone.0202868)

A

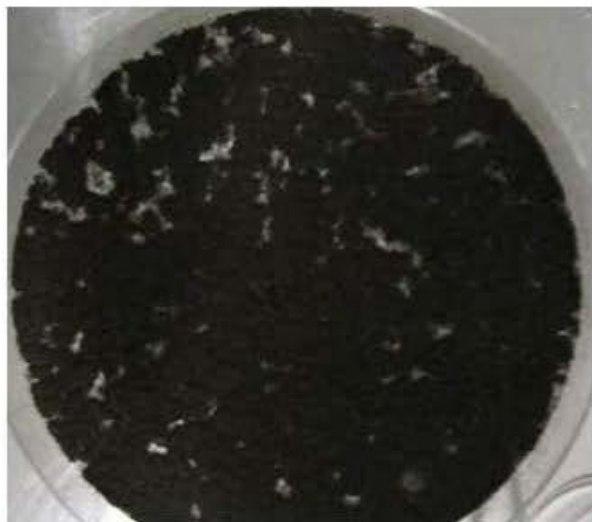

+ ANEp8-Cas9

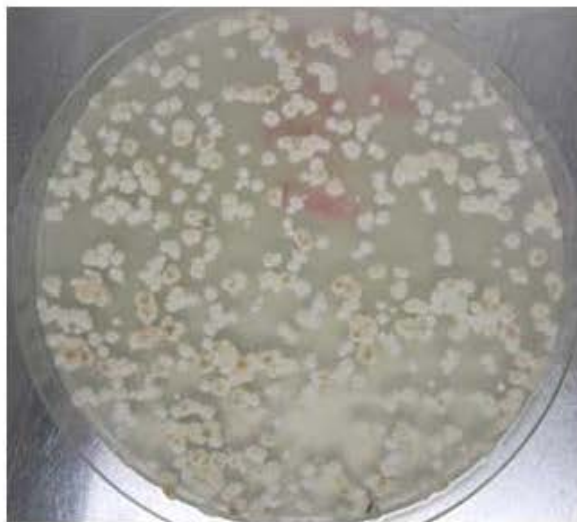

+ ANEp8-Cas9-gRNAalbA

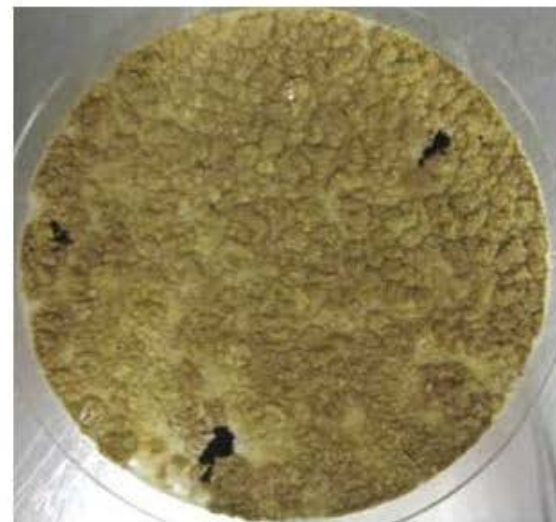

+ ANEp8-Cas9-gRNAolva

B

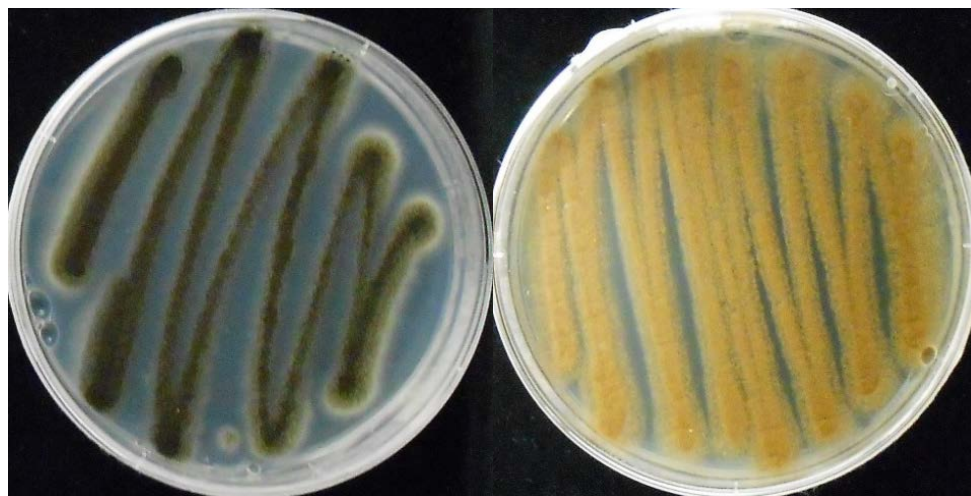

+ ANEp8-Cas9

+ ANEp8-Cas9-gRNAolva

Supplement: S1 Fig — A) From left to right are the transformation plates of A. niger N593 cells transformed with ANEp8-Cas9 plasmid (only expressed cas9), and ANEp8-Cas9-gRNA plasmid bearing tRNAPro1-driven gRNA to disrupt albA (98% efficiency) and olvA (95% efficiency) respectively. B) Growth phenotype of purified colonies of A. niger transformed with ANEp8-Cas9 and ANEp8-Cas9-gRNAolvA. (PDF) [file pone.0202868.s001.pdf]

**A**

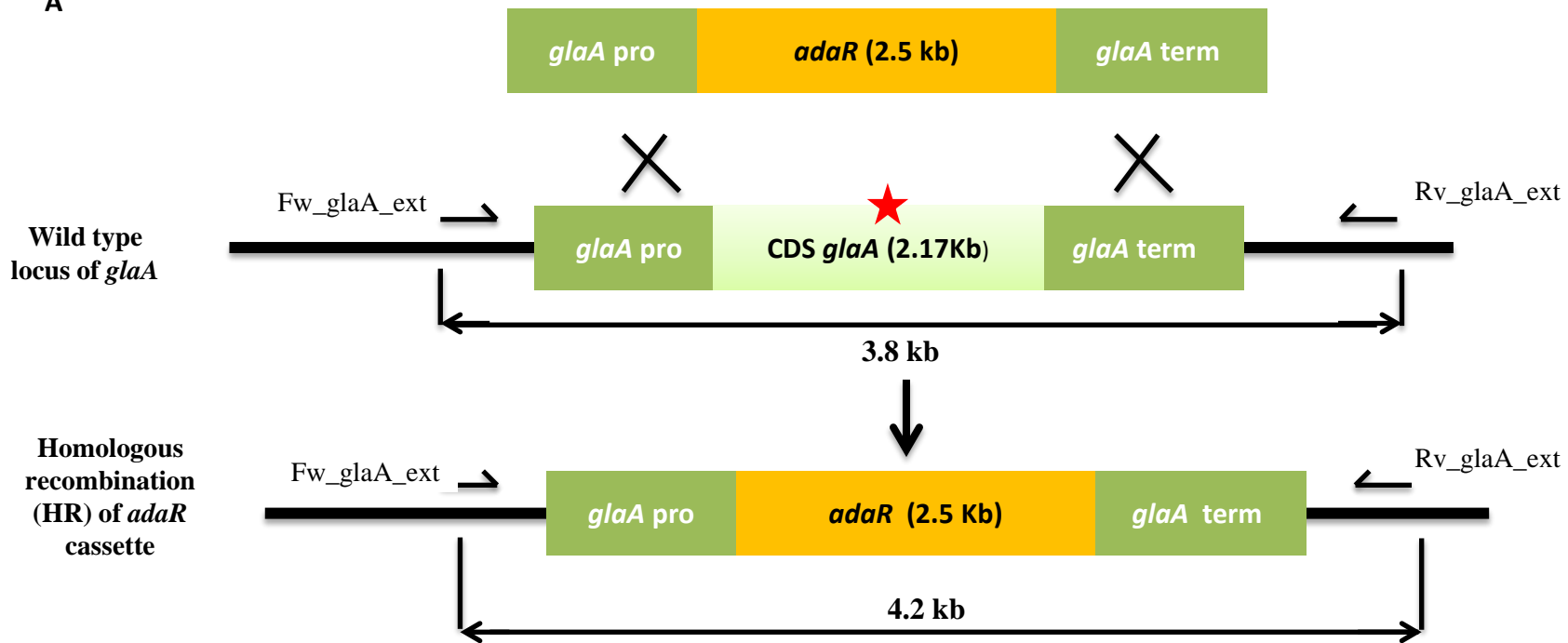

**B**

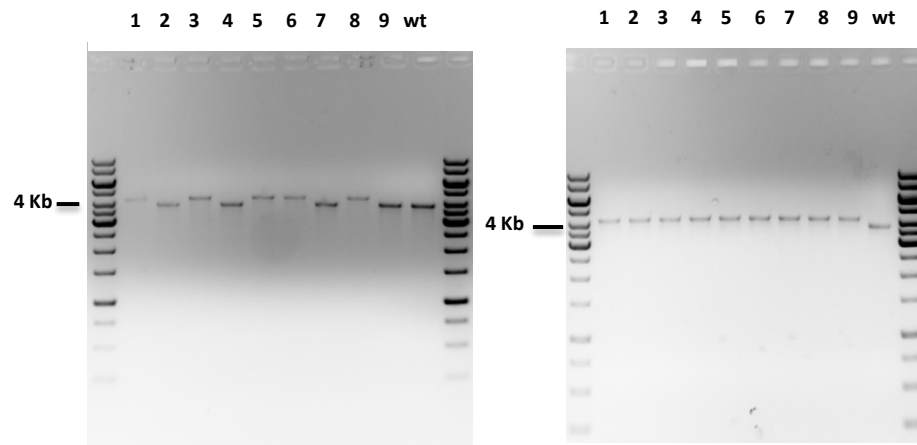

Supplement: S2 Fig — (A) Schematic illustration of targeted replacement by homologous recombination. (B) PCR screening for CRISPR/Cas9-mediated homologous integration among the nine-selected orange transformants from kusA+ (left) and kusA- (right) strains. The PCR was performed using their genomic DNA as template with primers outside of glaA locus. Replacement of glaA by adaR gave a 4.2 kb PCR fragment. Random integration of adaR cassette would leave the glaA locus intact, resulting in a 3.8 kb PCR fragment. (PDF) [file pone.0202868.s002.pdf]

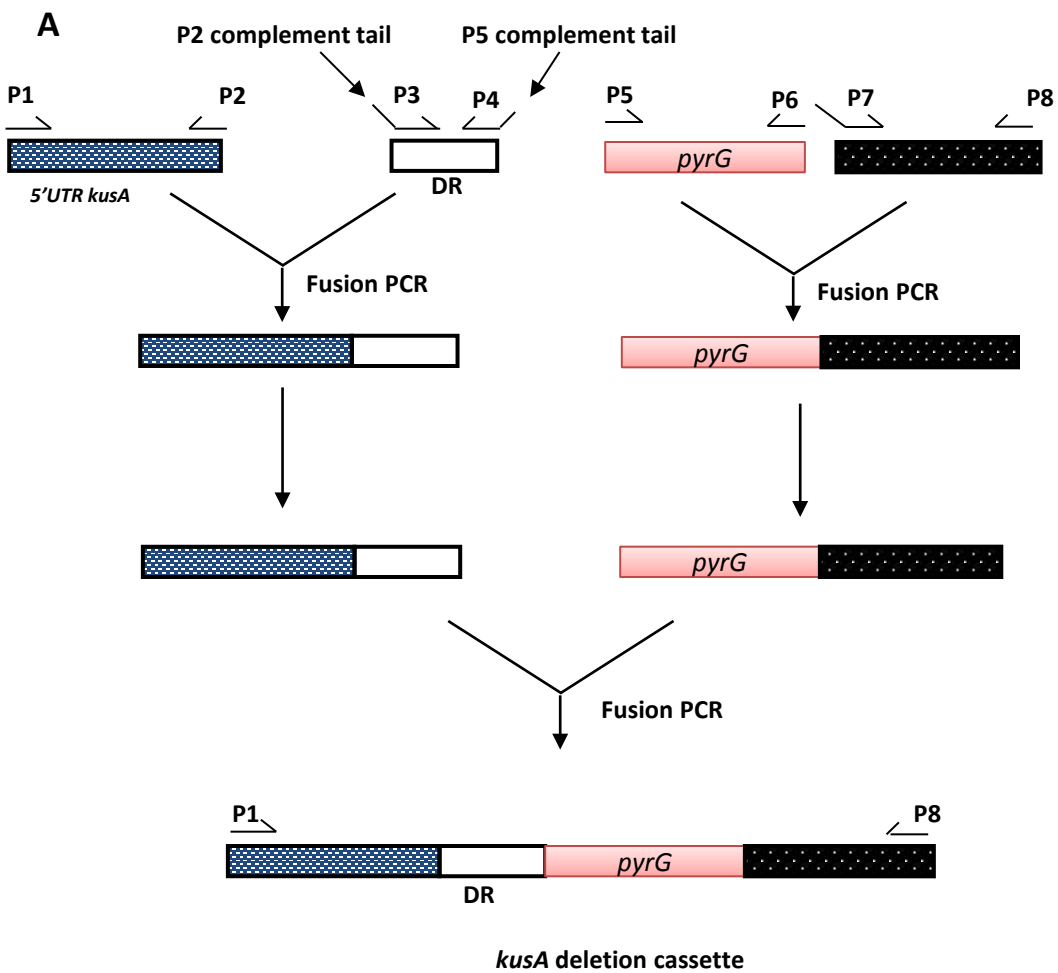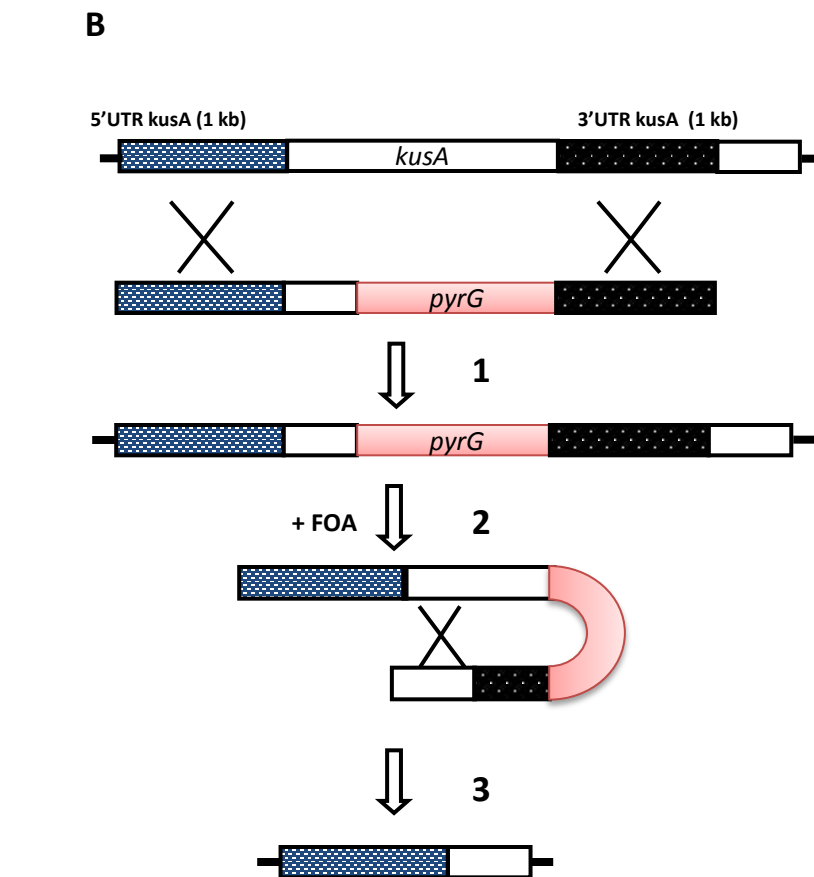

Loop out strategy of *pyrG* under 5' FOA and due to repeat sequences

Supplement: S3 Fig — (A) Fusions PCR to construct the kusA deletion cassette by including 500 bps repeat of the 3’ UTR in 5’ of the pyrG selection marker to facilitate the loop out of the selection marker. (B) Loop out strategy of the pyrG selection marker to generate ΔkusAΔpyrG strain for subsequent transformation with the same selection marker: (1) Homologous integration of the kusA deletion cassette to remove the kusA gene in A. niger; (2) Generation of ΔkusAΔpyrG genotype in presence of 5-FOA plus uridine to facilitate pyrG loop out (3). (PDF) [file pone.0202868.s003.pdf]

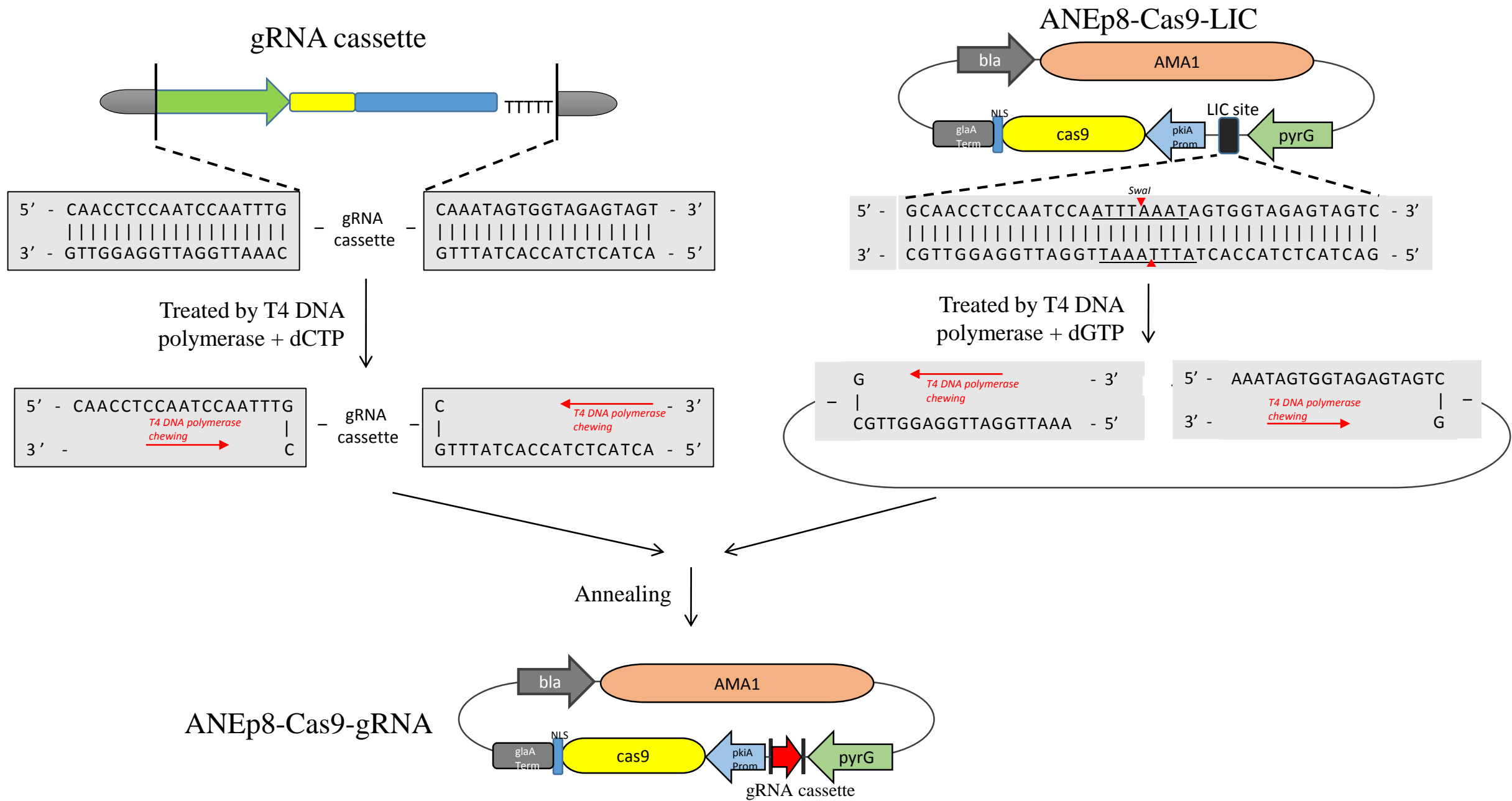

Supplement: S5 Fig — In ANEp8-Cas9 linearized vector, the single-strand 5' overhangs for LIC cloning were achieved by 3' →5' exonuclease activity of T4 DNA polymerase in the presence of dGTP. For gRNA cassette, the LIC tails were added to the ends via PCR, and the reverse complementing overhangs were generated by the same T4 DNA polymerase treatment process with dCTP. Through base pairing, plasmid ANEp8-Cas9-gRNA was assembled by annealing the complementary sequences between gRNA insert and plasmid vector. The complementary LIC sequences in the gRNA insert and ANEp8-Cas9 vector are shown in the figure. (PDF) [file pone.0202868.s005.pdf]
