# Supplementary material for: Efficient genome editing using tRNA promoter-driven CRISPR/Cas9 gRNA in Aspergillus niger
Source: PLoS One. 2018 Aug 24;13(8):e0202868. doi: 10.1371/journal.pone.0202868 (PMC6108506; doi:10.1371/journal.pone.0202868)
Supplement: S4 Fig — (A) Screening of transformants for ΔkusApyrG+ genotype with primers binding externally to the kusA locus and primers binding within the pyrG selection marker. Two PCR bands with size of 3 kb and 2.5 kb respectively are expected by using primer pairs P1_ext/P6_int and P5_int/P8_ext. Transformants 3, 6, 9 and 13 have the deletion cassette integrated in the kusA locus and have ΔkusApyrG+ genotype, when transformants 4, 5, 7, 8, 10, 11, 12 and 14 have partial integration of the deletion cassette. For transformants lacking pyrG, the primers are not able to bind and amplify the gDNA of the parental strain (PS) NRRL2270pyrG-. (B) Screening for transformants with pyrG loop out. Spores of transformant 3 (NRRL2270ΔkusApyrG+) have been plated on minimal medium containing 5-FOA and uridine for 5 days. Colonies growing on 5-FOA are selected for screening pyrG selection marker loop out phenotype by PCR. A correct loop out of the pyrG marker is characterized by a PCR giving a band size of 2.2 kb, while a strain failed in pyrG loop out will display a band of 4 kb. Transformant 3’ with the correct loop out of the pyrG selection marker is NRRL2270ΔkusAΔpyrG and has been selected for further work. (C) Growth comparison of NRRL2270ΔpyrG and NRRL2270ΔkusAΔpyrG. One million spores of strains NRRL2270ΔkusAΔpyrG and NRRL2270ΔpyrG were plated on the uridine-contained minimal or complete medium, and incubated at 30°C for 3 days. (PDF) [file pone.0202868.s004.pdf]

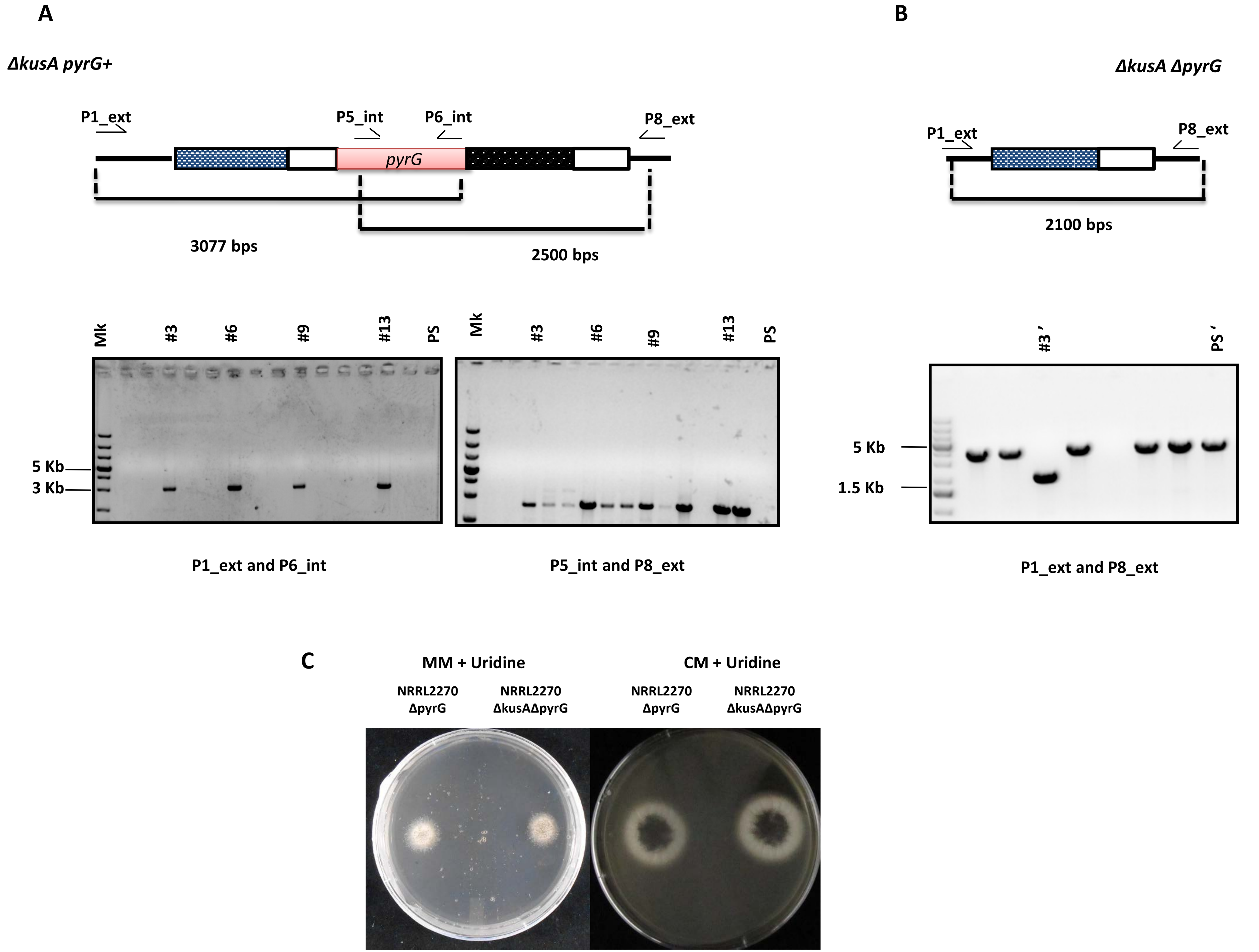
A
ΔkusA pyrG+
P1_ext
P5_int
P6_int
P8_ext
pyrG
3077 bps
2500 bps
Mk
#3
#6
#9
#13
PS
5 Kb
3 Kb
P1_ext and P6_int
P5_int and P8_ext
B
ΔkusA ΔpyrG
P1_ext
P8_ext
2100 bps
#3 ´
PS ´
5 Kb
1.5 Kb
P1_ext and P8_ext
C
MM + Uridine
CM + Uridine
NRRL2270
ΔpyrG
NRRL2270
ΔkusAΔpyrG
NRRL2270
ΔpyrG
NRRL2270
ΔkusAΔpyrG
