# Supplementary material for: Efficient genome editing using tRNA promoter-driven CRISPR/Cas9 gRNA in Aspergillus niger
Source: PLoS One. 2018 Aug 24;13(8):e0202868. doi: 10.1371/journal.pone.0202868 (PMC6108506; doi:10.1371/journal.pone.0202868)
Supplement: S3 Table — (DOCX) [file pone.0202868.s008.docx]

**Table S3**: CRISPR guide sequences applied in gene editing

| **Guide ID** | **Targeted gene** | **Genomic target sequence (5'>3')** | **PAM** | **Direction** | **Location** | ***On-target activity score** | ***Off-target score (%)** |
| --- | --- | --- | --- | --- | --- | --- | --- |
| albA-G1 | *albA* | AGTGGGATCTCAAGAACTAC | TGG | Forward | 3764-3786 | 0.292 | 100% |
| olvA-G1 | *olvA* | GTACGGCTACTCCAACCCGG | AGG | Forward | 933-955 | 0.905 | 100% |
| glaA-G1 | *glaA* | CGACGGTGACTGACACCTGG | GGG | Forward | 1898-1920 | 0.851 | 100% |

*On-target and off-target scores were evaluated by Geneious software. On-target scores are between 0 and 1, with a higher score denoting higher expected activity. Off-target scores are between 0 and 100%, with a higher score denoting less off-target activity
