# Supplementary material for: Efficient genome editing using tRNA promoter-driven CRISPR/Cas9 gRNA in Aspergillus niger
Source: PLoS One. 2018 Aug 24;13(8):e0202868. doi: 10.1371/journal.pone.0202868 (PMC6108506; doi:10.1371/journal.pone.0202868)
Supplement: S5 Table — (PDF) [file pone.0202868.s010.pdf]

Sequence of ANEp8_Cas9

AMA1, pyrG, pKi promoter, Cas9, NLS, GlaA terminator

GGCTGTACTTACGGGCCCTCGCCATTGTCCTGATCATTTCCAGCTTCACCCTCGTTGCTGCAAAGTAGTTAGTGACTAGTCAAGGACTAGTTGAAATGGGAGAAGAAACTCACGAATTCTCGACTCCCTTAGTATTGT
GGTCCTTGGACTTGGTGCTGCTATATATTAGCTAATACACTAGTTAGACTCACAGAAACTTACGCAGCTCGCTTGCGCTTCTTGGTAGGAGTCGGGGTTGGGAGAACAGTGCCTTCAAACAAGCCTTCATACCATGCT
ACTTGACTAGTCAGGGACTAGTCACCAAGTAATCTAGATAGGACTTGCCTTTGGCCTCCATCAGTTCCTTCATAGTGGGAGGACCATTGTGCAATGTAAACTCCATGCCGTGGGAGTTCTTGTCCTTCAAGTGCTTGA
CCAATATGTTTCTGTTGGCAGAGGGAACCTGTCAACTAGTTAATAACTAGTCAGAAACTATGATAGCAGTAGACTCACTGTACGCTTGAGGCATCCCTTCACTCGGCAGTAGACTTCATATGGATGGATATCAGGCA
CGCCATTGTCGTCCTGTGGACTAGTCAGTAACTAGGCTTAAAGCTAGTCGGGTCGGCTTACTATCTTGAAATCCGGCAGCGTAAGCTCCCCGTCCTTAACTGCCTCGAGATAGTGACAGTACTCTGGGGACTTTCGGA
GATCGTTATCGTTATCGCGAATGCTCGGCATACTAACTGTTGACTAGTCTTGGACTAGTCCCGAGCAAAAAGGATTGGAGGAGGAGGAGGAAGGTGAGAGTGAGACAAAGAGCGAAATAAGAGCTTCAAAGGCT
ATCTCTAAGCAGTATGAAGGTTAAGTATCTAGTTCTTGACTAGATTTAAAGAGATTTCGACTAGTTATGTACCTGGAGTTTGGATATAGGAATGTGTTGTGGTAACGAAATGTAAGGGGGAGGAAAGAAAAAGTC
GTCAAGAGGTAACTCTAAGTCGGCCATTCCTTTTTGGGAGGCGCTAACCATAAACGGCATGGTCGACTTAGAGTTAGCTCAGGGAATTTAGGGAGTTATCTGCGACCACTGGACTCTCACCGTTTCCATTGGGGTAC
TAACATAGCCATCAAATGCCGGCCCGTGTTTAATCTCCTCGAGATGGCTGGGCTCACATGCGTTCTACTCAAAAACTCTTATACCTAGGCCCTCTCCCAATCGTGTATGTTTGGACCTTCCTCTCTCACACTTTGGATAA
CATCAAGCATACTGCAGATTGGTACCTAGTTAACAAGGCACATCATGCTATCACTTCCAACACTCTTGCTGAGCGCGTTCTGCCTTGCCTTTCTGGTTCTACGAAGTATCCAGTCCCGCCTGCAACGCCGCAGAAACGC
CAAAAGGTGGGGTTGTCAACCGCCAGCTAGAGCTCCATCGGGATTCTTTGGCATTCCGCCGTTCCTCGGTGGTCGCAGATAACTCCCTAAATTCCCTGAGCTAACTCTAAGTCGACCATGCCGTTTATGGTTAGCGCC
TCCCAAAAAGGAATGGCCGACTTAGAGTTACCTCTTGACGACTTTTTCTTTCCTCCCCCTTACATTTCGTTACCACAACACATTCCTATATCCAAACTCCAGGTACATAACTAGTCGAAATCTCTTTAAATCTAGTCAAG
AACTAGATACTTAACCTTCATACTGCTTAGAGATAGCCTTTGAAGCTCTTATTTCGCTCTTTGTCTCACTCTCACCTTCCTCCTCCTCCTCCAATCCTTTTTGCTCGGGACTAGTCCAAGACTAGTCAACAGTTAGTATGCC
GAGCATTCGCGATAACGATAACGATCTCCGAAAGTCCCCAGAGTACTGTCACTATCTCGAGGCAGTTAAGGACGGGGAGCTTACGCTGCCGGATTTCAAGATAGTAAGCCGACCCGACTAGCTTTAAGCCTAGTTA
CTGACTAGTCCACAGGACGACAATGGCGTGCCTGATATCCATCCATATGAAGTCTACTGCCGAGTGAAGGGATGCCTCAAGCGTACAGTGAGTCTACTGCTATCATAGTTTCTGACTAGTTATTAACTAGTTGACAG
GTTCCCTCTGCCAACAGAAACATATTGGTCAAGCACTTGAAGGACAAGAACTCCCACGGCATGGAGTTTACATTGCACAATGGTCCTCCCACTATGAAGGAACTGATGGAGGCCAAAGGCAAGTCCTATCTAGATT
ACTTGGTGACTAGTCCCTGACTAGTCAAGTAGCATGGTATGAAGGCTTGTTTGAAGGCACTGTTCTCCCAACCCCGACTCCTACCAAGAAGCGCAAGCGAGCTGCGTAAGTTTCTGTGAGTCTAACTAGTGTATTAG
CTAATATATAGCAGCACCAAGTCCAAGGACCACAATACTAAGGGAGTCGAGAATTCGTGAGTTTCTTCTCCCATTTCAACTAGTCCTTGACTAGTCACTAACTACTTTGCAGCAACGAGGGTGAAGCTGGAAATGAT
CAGGACAATGGCGAGGGCCCGTAAGTACAGCCATTCAATGCAGACTAGTTGCTAACTAATCTGTGACTAGTTCAAGTGGTCCGTACGCCGTGCATACCCCTGTGACTGGTAGGAATTTGAGCAAGCCTGTCTTGCCG
CGCGATGAGAAAGGAAAGGCAAGTTACATTCAGCCCCGTACCTAGGATCAGTGCTAATTTATAACCTCTAGCCACTCTTTATGGCAGATCCGCCGTGAGGGTAGCAAGGCAGCTAAATCAGCTGGTGAGAAAGGA
ACCATACCCTGCAAGACCTGTCGCAACGCAAAGGGCAAAGGTAAGCTATCCAAGCTAGTTTGGGACTAGATTCTAACTAGTCTCAGCACCGTGTGGTTCAAAGCCATATTGCGAGTTTTGGCGCTTTTTCTCATCGAT
TGACGAGGCAAAGGGAGCGAGTATGCAACCTCGTAAGTCAGACTCAGACAAAGACCAACTAGTTATTGACCAGTCTTCTAGAAGGCTCTGTTGTGGATCTTGAGGCCCTGGAGAGTTCCTCCAACAATCCGGAGAC
AAGCAAGTCCTCGTCGGACTAGTCACTAACTAGACTCTAACTAGTTGCAGACATGGATAATGCAAAAGAGACAAGCAATGAAGAAAGTGGTAAGACATTTCTCCTTTGTGGTTCTGGACTAGTCTTTGACTAGTCAC
AGTCTTAAACAAGGAAAATGAGCATGAAAATGAGGAGGAAAAGGCTGCTGAGCCCGAGGAAGTGCAGGGTGATGGCAGACATGGTAGGTTAATACCTTGTTAGTTATTGCTAGTCACTGACTAGTCAATAACTA
GTCTCTGAACACCTTGCAATCACTCCGTTTGCGCAGCTGAACAGTGGTGAGGATAATAGTAGTAAGTTATTCTAGCTTCAGAGTTATAGGAGACTAGATACTAACTAGTATTAGTTGCAACTAACCTGGATCTCAGA
GACTTTGGCCTCAATCTAGAATCTATCTAGTTGTCAACTAGACTGTGGTATCATTGTCTTTTATTTTCCTAGTCCTGGAACTAGCTTCTAACTAGTCTCCCTAATATGTGGCTGTCTTGTTTTTTTTTTTTGTTTCCCTACCC
GGATATCTAGTCCCCTTCTAGGTTCTGTTAACCTCTCGGGCTCTGATTTAGTTTAACGCAAACCTGAGATTAGTTTCTAACTAGTCTCTAGGTTTTCTATCCACCTTTAATTGTAATAATAAATACAAGCAACGTTTATA
CGTCAAAAGCATTTATAAACTTTTACCCTAAAGTAGCTTGCTTGTGTGTTTAGTTTATAATTAGTCTCTTATTAATTTGATGTAGGTAAGCCCGCCACAAATATATATTTTTACAAGATACCGTGGAAAAACTTCGTGCT
ATCACAAAACAGTATACAAAAAATAAGCTTGCGGCCGCGCAACTTCCTCGAGAACGCGCCGCAGACAATGCTCTCTATCCTGGTGGCAGGCGTCAAGTACCCAGAGGCAGCAGCGGGCTTAGGAGCGGCCTGGGTTG
TTCTCCGCACCCTCTACATGCTGGGCTATATTTATAGCGACAAGCCGAACGGCACCGGCAGGTACAATGGTTCGCTGTACTTGCTTGCGCAAGCGGGTCTTTGGGGATTGAGCGCATTTGGTGTTGCAAAGGATTTGAT
GTAAATGTAGTCGACATCTTAGCACAGAGGGGAGAGTTGATAAAATGTGGTCTGTTTGAATGATAGTCGGGTTCGTGACCTATATTCGTGATAGTGGAGATAGGTCTGCGCCTATCTTATCGGGCCGGAGCAAAAATTC
CACCGCAGCGGGGTGAGTTTTCGTTATACAGCCATCCCACTTCCAGCTTCAAATTGTCAGTTTAATCCAGCCCAATTCAATCATTGGAGAACCGCCATCATGTCTTCGAAGTCCCACCTCCCCTACGCAATTCGCGCAACC
AACCATCCCAACCCTTTAACATCTAAACTCTTCTCCATCGCCGAGGAGAAGAAAACCAACGTCACCGTCTCCGCAGACGTTACTACTTCCGCCGAGCTCCTCGATCTTGCTGACCGTACATCCTGCACCAATGCCCCTCCA
GGATAACAAATAGCTGATGCGTAGTGAGTACAGGCCTAGGCCCCTATATCGCAGTTCTGAAAACCCACATCGACATCCTCACCGATCTCACCCCGTCGACCCTTTCCTCGCTCCAATCCCTCGCGACAAAGCACAACTTCC
TCATCTTTGAGGACCGCAAGTTCATCGACATCGGCAACACCGTGCAAAAGCAGTACCACGGTGGCGCTCTCCGCATCTCCGAATGGGCACACATCATCAACTGCGCCATCCTGCCGGGCGAAGGGATCGTCGAGGCCCT
CGCACAGACAACCAAGTCTCCTGACTTTAAAGACGCGAATCAACGAGGTCTCCTGATTCTTGCCGAGATGACGAGTAAGGGATCTCTTGCGACAGGGGAGTCACAGGCACGCTCGGTTGAGTACGCGCGGAAGTATAA
GGGGTTTGTGATGGGATTCGTGAGTACAAGGGCGTTGAGTGAGGTGCTGCCCGAACAGAAAGAGGAGAGCGAGGATTTTGTCGTCTTTACGACTGGGGTGAATCTGTCGGATAAGGGGGATAAGCTGGGGCAGCAG
TATCAGACACCTGGGTCGGCGGTTGGGCGAGGTGCGGACTTTATCATTGCGGGTAGGGGCATCTATAAGGCGGACGATCCAGTCGAGGCGGTTCAGAGGTACCGGGAGGAAGGCTGGAAAGCTTACGAGAAAAGA
GTTGGACTTTGAGTGTGAGTGGAAATGTGTAACGGTATTGACTAAAAGGGGACGTCCCACATCTGATGCCATTGGCGGAGGGGTCCGGACGGTCAGGAACTTAGCCTTATGAGATGAATGATGGACGTGTCTGGCCT
CGGAAAAGGATATATGGGGATCATAATAGTACTAGCCATATTAATGAAGGGCATATACCACGCGACTACTCTACCACTATTTAAATTGGATTGGAGGTTGCGGCGCGCCGTTTAAACAAAGCAGCACGCGTAAATGGA
AGAGAAAACCTCCGAGTACTTACTTAGGGCCCTGTCTACTGGCCAGAGTCTCGTCCTCTATTCACTATGATAAATTACCCACTGGACAAAAAAATAAAATAAAATAAAAATAAAAAGGGAGACAGCTTCTCCATCAACTG
GCAACTGGGTCCGTCCGAGCAGAGCAAAATTCAGCCTTATGGGTTCCGATGGAGTCAGGGAAATAGTTCTTGCGAAGGGCATTGGGCTTTTTTGCGAGGAGAAAATTCAGCACCGACAAAGCATCCGAAATCCGACCT
CGCTAGGAGAGAATGGATCCGCGACGATGTGGGGTCAACTGGACAGAGTGAGAGGGTATCATGTGGTCCTGCCAGATACTTCGCAGAATGTTGTGTGGGTGTCTGATTGTGGCTTGGGCGTGAATTGCTTTTGGTCTT
CCCAACCAATTATTATTGCATGCGGCGTATGAATGCCTGAGATGCGCGGAGGGAAGGTGCCTGAGGATGTAGTGGACAAATGCTGCTGATCGCTGGGCGGAAACCCTTGGCTGACCAGTGAAAAGAGCGGACGGAG
GCAGCAGGTGTATCTACGATCAAAGAATAGTAGCAAAGCAGTGAAAGGTGGATCACCCAGCAAATAATTGAGTTTTGATACCCAGCGATAGTGCCGGGGGGGAGAAAAAGTCATTAATAATGGGAATTATGTAGGCG
ATGGGAAGTGTGATTGTAACTACTCCGTAGCTGGAGGCACAACTAACAAGCCAGCTCTCAACCCGCGGGGAACCGACCGACAGATAAAAAAAAAGCGTCCCAAAGCAGGAATCCCACCAAAAAGGGCCGATCCAGCCA
ATCACCGCCGCCAACATTTTTCCTTCCCGGGCACCCCTCCTCTAGTCCACCATCTCTCTCTTCTCTCGCTCACCGGCCCCGTCTTTTCCTTCCCTATTATCTCTCCCTCTTCTCCTCCCTTCTCTCCCTCCATTCTTTCTCCCATCT
TCATCACTCCCTTCTCTTCTGTCTTCCCCCCGGTTCAGTAGAGATCAATCAGCTAGCTTAATTAAATGGACAAGAAGTACTCCATTGGGCTCGCTATCGGCACAAACAGCGTCGGCTGGGCCGTCATTACGGACGAGTAC
AAGGTGCCGAGCAAAAAATTCAAAGTTCTGGGCAATACCGATCGCCACAGCATAAAGAAGAACCTCATTGGCGCCCTCCTGTTCGACTCCGGGGAGACGGCCGAAGCCACGCGGCTCAAAAGAACAGCACGGCGCAG
ATATACCCGCAGAAAGAATCGGATCTGCTACCTGCAGGAGATCTTTAGTAATGAGATGGCTAAGGTGGATGACTCTTTCTTCCATAGGCTGGAGGAGTCCTTTTTGGTGGAGGAGGATAAAAAGCACGAGCGCCACCC
AATCTTTGGCAATATCGTGGACGAGGTGGCGTACCATGAAAAGTACCCAACCATATATCATCTGAGGAAGAAGCTTGTAGACAGTACTGATAAGGCTGACTTGCGGTTGATCTATCTCGCGCTGGCGCATATGATCAAA
TTTCGGGGACACTTCCTCATCGAGGGGGACCTGAACCCAGACAACAGCGATGTCGACAAACTCTTTATCCAACTGGTTCAGACTTACAATCAGCTTTTCGAAGAGAACCCGATCAACGCATCCGGAGTTGACGCCAAAG
CAATCCTGAGCGCTAGGCTGTCCAAATCCCGGCGGCTCGAAAACCTCATCGCACAGCTCCCTGGGGAGAAGAAGAACGGCCTGTTTGGTAATCTTATCGCCCTGTCACTCGGGCTGACCCCCAACTTTAAATCTAACTTC
GACCTGGCCGAAGATGCCAAGCTTCAACTGAGCAAAGACACCTACGATGATGATCTCGACAATCTGCTGGCCCAGATCGGCGACCAGTACGCAGACCTTTTTTTGGCGGCAAAGAACCTGTCAGACGCCATTCTGCTGA
GTGATATTCTGCGAGTGAACACGGAGATCACCAAAGCTCCGCTGAGCGCTAGTATGATCAAGCGCTATGATGAGCACCACCAAGACTTGACTTTGCTGAAGGCCCTTGTCAGACAGCAACTGCCTGAGAAGTACAAGG
AAATTTTCTTCGATCAGTCTAAAAATGGCTACGCCGGATACATTGACGGCGGAGCAAGCCAGGAGGAATTTTACAAATTTATTAAGCCCATCTTGGAAAAAATGGACGGCACCGAGGAGCTGCTGGTAAAGCTTAACA
GAGAAGATCTGTTGCGCAAACAGCGCACTTTCGACAATGGAAGCATCCCCCACCAGATTCACCTGGGCGAACTGCACGCTATCCTCAGGCGGCAAGAGGATTTCTACCCCTTTTTGAAAGATAACAGGGAAAAGATTGA
GAAAATCCTCACATTTCGGATACCCTACTATGTAGGCCCCCTCGCCCGGGGAAATTCCAGATTCGCGTGGATGACTCGCAAATCAGAAGAGACCATCACTCCCTGGAACTTCGAGGAAGTCGTGGATAAGGGGGCCTCT
GCCCAGTCCTTCATCGAAAGGATGACTAACTTTGATAAAAATCTGCCTAACGAAAAGGTGCTTCCTAAACACTCTCTGCTGTACGAGTACTTCACAGTTTATAACGAGCTCACCAAGGTCAAATACGTCACAGAAGGGAT
GAGAAAGCCAGCATTCCTGTCTGGAGAGCAGAAGAAAGCTATCGTGGACCTCCTCTTCAAGACGAACCGGAAAGTTACCGTGAAACAGCTCAAAGAAGACTATTTCAAAAAGATTGAATGTTTCGACTCTGTTGAAATC
AGCGGAGTGGAGGATCGCTTCAACGCATCCCTGGGAACGTATCACGATCTCCTGAAAATCATTAAAGACAAGGACTTCCTGGACAATGAGGAGAACGAGGACATTCTTGAGGACATTGTCCTCACCCTTACGTTGTTTG
AAGATAGGGAGATGATTGAAGAACGCTTGAAAACTTACGCTCATCTCTTCGACGACAAAGTCATGAAACAGCTCAAGAGGCGCCGATATACAGGATGGGGGCGGCTGTCAAGAAAACTGATCAATGGGATCCGAGAC
AAGCAGAGTGGAAAGACAATCCTGGATTTTCTTAAGTCCGATGGATTTGCCAACCGGAACTTCATGCAGTTGATCCATGATGACTCTCTCACCTTTAAGGAGGACATCCAGAAAGCACAAGTTTCTGGCCAGGGGGACA
GTCTTCACGAGCACATCGCTAATCTTGCAGGTAGCCCAGCTATCAAAAAGGGAATACTGCAGACCGTTAAGGTCGTGGATGAACTCGTCAAAGTAATGGGAAGGCATAAGCCCGAGAATATCGTTATCGAGATGGCCC
GAGAGAACCAAACTACCCAGAAGGGACAGAAGAACAGTAGGGAAAGGATGAAGAGGATTGAAGAGGGTATAAAAGAACTGGGGTCCCAAATCCTTAAGGAACACCCAGTTGAAAACACCCAGCTTCAGAATGAGAA
GCTCTACCTGTACTACCTGCAGAACGGCAGGGACATGTACGTGGATCAGGAACTGGACATCAATCGGCTCTCCGACTACGACGTGGATCATATCGTGCCCCAGTCTTTTCTCAAAGATGATTCTATTGATAATAAAGTGT
TGACAAGATCCGATAAAAATAGAGGGAAGAGTGATAACGTCCCCTCAGAAGAAGTTGTCAAGAAAATGAAAAATTATTGGCGGCAGCTGCTGAACGCCAAACTGATCACACAACGGAAGTTCGATAATCTGACTAAG
GCTGAACGAGGTGGCCTGTCTGAGTTGGATAAAGCCGGCTTCATCAAAAGGCAGCTTGTTGAGACACGCCAGATCACCAAGCACGTGGCCCAAATTCTCGATTCACGCATGAACACCAAGTACGATGAAAATGACAAA
CTGATTCGAGAGGTGAAAGTTATTACTCTGAAGTCTAAGCTGGTCTCAGATTTCAGAAAGGACTTTCAGTTTTATAAGGTGAGAGAGATCAACAATTACCACCATGCGCATGATGCCTACCTGAATGCAGTGGTAGGCA
CTGCACTTATCAAAAAATATCCCAAGCTTGAATCTGAATTTGTTTACGGAGACTATAAAGTGTACGATGTTAGGAAAATGATCGCAAAGTCTGAGCAGGAAATAGGCAAGGCCACCGCTAAGTACTTCTTTTACAGCAAT
ATTATGAATTTTTTCAAGACCGAGATTACACTGGCCAATGGAGAGATTCGGAAGCGACCACTTATCGAAACAAACGGAGAAACAGGAGAAATCGTGTGGGACAAGGGTAGGGATTTCGCGACAGTCCGGAAGGTCCT
GTCCATGCCGCAGGTGAACATCGTTAAAAAGACCGAAGTACAGACCGGAGGCTTCTCCAAGGAAAGTATCCTCCCGAAAAGGAACAGCGACAAGCTGATCGCACGCAAAAAAGATTGGGACCCCAAGAAATACGGCG
GATTCGATTCTCCTACAGTCGCTTACAGTGTACTGGTTGTGGCCAAAGTGGAGAAAGGGAAGTCTAAAAAACTCAAAAGCGTCAAGGAACTGCTGGGCATCACAATCATGGAGCGATCAAGCTTCGAAAAAAACCCCA
TCGACTTTCTCGAGGCGAAAGGATATAAAGAGGTCAAAAAAGACCTCATCATTAAGCTTCCCAAGTACTCTCTCTTTGAGCTTGAAAACGGCCGGAAACGAATGCTCGCTAGTGCGGGCGAGCTGCAGAAAGGTAACG
AGCTGGCACTGCCCTCTAAATACGTTAATTTCTTGTATCTGGCCAGCCACTATGAAAAGCTCAAAGGGTCTCCCGAAGATAATGAGCAGAAGCAGCTGTTCGTGGAACAACACAAACACTACCTTGATGAGATCATCGA
GCAAATAAGCGAATTCTCCAAAAGAGTGATCCTCGCCGACGCTAACCTCGATAAGGTGCTTTCTGCTTACAATAAGCACAGGGATAAGCCCATCAGGGAGCAGGCAGAAAACATTATCCACTTGTTTACTCTGACCAAC
TTGGGCGCGCCTGCAGCCTTCAAGTACTTCGACACCACCATAGACAGAAAGCGGTACACCTCTACAAAGGAGGTCCTGGACGCCACACTGATTCATCAGTCAATTACGGGGCTCTATGAAACAAGAATCGACCTCTCTC
AGCTCGGTGGAGACAGCAGGGCTGACCCCAAGAAGAAGAGGAAGGTGTAAGGCCGGCCCGACCGCGACGGTGACTGACACCTGGCGGTAGACAATCAATCCATTTCGCTATAGTTAAAGGATGGGGATGAGGGCAA
TTGGTTATATGATCATGTATGTAGTGGGTGTGCATAATAGTAGTGAAATGGAAGCCAAGTCATGTGATTGTAATCGACCGACGGAATTGAGGATATCCGGAAATACAGACACCGTGAAAGCCATGGTCTTTCCTTCGTG

TAGAAGACCAGACAGACAGTCCCTGATTTACCCTGCACAAAGCACTAGAAAATTAGCATTCCATCCTTCTCTGCTTGCTCTGCTGATATCACTGTCATTCAATGCATAGCCATGAGCTCATCTTAGATCCAAGCACGTAATT
CCATAGCCGAGGTCCACAGTGGAGCAGCAACATTCCCCATCATTGCTTTCCCCAGGGGCCTCCCAACGACTAAATCAAGAGTATATCTCTACCGTCCAATAGATCGTCTTCGCTTCAAAATCTTTGACAATTCCAAGAGGG
TCCCCATCCATCAAACCCAGTTCAATAATAGCCGAGATGCATGGTGGAGTCAATTAGGCAGTATTGCTGGAATGTCGGGGCCAGTTCCGGGTGGTCATTGGCCGCCTGTGATGCCATCTGCCACTAAATCCGATCATTG
ATCCACCGCCCACGAGGGCGTCTTTGCTTTTTGCGCGGCGTCCAGGTTCAACTCTCTCTGCAGCTCCAGTCCAACGCTGACTGACTAGTTTACCTACTGGTCTGATCGGCTCCATCAGAGCTATGGCGTTATCCCGTGCCG
TTGCTGCGCAATCGCTATCTTGATCGCAACCTTGAACTCACTCTTGTTTTAATAGTGATCTTGGTGACGGAGTGTCGGTGAGTGACAACCAACATCGTGCAAGGGAGATTGATACGGAATTGTCGCTCCCATCATGATGT
TCTTGCCGGCTTTGTTGGCCCTATTCGTGGGATCGATGCCCTCCTGTGCAGCAGCAGGTACTGCTGGATGAGGAGCCATCGGTCTCTGCACGCAAACCCAACTTCCTCTTCATTCTCACGGATGATCAGGATCTCCGGAA
TATTCGCGGACCTGCAGGCATGCAAGCTTGGCACTGGCCGTCGTTTTACAACGTCGTGACTGGGAAAACCCTGGCGTTACCCAACTTAATCGCCTTGCAGCACATCCCCCTTTCGCCAGCTGGCGTAATAGCGAAGAGG
CCCGCACCGATCGCCCTTCCCAACAGTTGCGCAGCCTGAATGGCGAATGGCGCCTGATGCGGTATTTTCTCCTTACGCATCTGTGCGGTATTTCACACCGCATATGGTGCACTCTCAGTACAATCTGCTCTGATGCCGCAT
AGTTAAGCCAGCCCCGACACCCGCCAACACCCGCTGACGCGCCCTGACGGGCTTGTCTGCTCCCGGCATCCGCTTACAGACAAGCTGTGACCGTCTCCGGGAGCTGCATGTGTCAGAGGTTTTCACCGTCATCACCGAA
ACGCGCGAGACGAAAGGGCCTCGTGATACGCCTATTTTTATAGGTTAATGTCATGATAATAATGGTTTCTTAGCAGGTGGCACTTTTCGGGGAAATGTGCGCGGAACCCCTATTTGTTTATTTTTCTAAATACATTCAAAT
ATGTATCCGCTCATGAGACAATAACCCTGATAAATGCTTCAATAATATTGAAAAAGGAAGAGTATGAGTATTCAACATTTCCGTGTCGCCCTTATTCCCTTTTTTGCGGCATTTTGCCTTCCTGTTTTTGCTCACCCAGAAA
CGCTGGTGAAAGTAAAAGATGCTGAAGATCAGTTGGGTGCACGAGTGGGTTACATCGAACTGGATCTCAACAGCGGTAAGATCCTTGAGAGTTTTCGCCCCGAAGAACGTTTTCCAATGATGAGCACTTTTAAAGTTCT
GCTATGTGGCGCGGTATTATCCCGTATTGACGCCGGGCAAGAGCAACTCGGTCGCCGCATACACTATTCTCAGAATGACTTGGTTGAGTACTCACCAGTCACAGAAAAGCATCTTACGGATGGCATGACAGTAAGAGAA
TTATGCAGTGCTGCCATAACCATGAGTGATAACACTGCGGCCAACTTACTTCTGACAACGATCGGAGGACCGAAGGAGCTAACCGCTTTTTTGCACAACATGGGGGATCATGTAACTCGCCTTGATCGTTGGGAACCGG
AGCTGAATGAAGCCATACCAAACGACGAGCGTGACACCACGATGCCTGTAGCAATGGCAACAACGTTGCGCAAACTATTAACTGGCGAACTACTTACTCTAGCTTCCCGGCAACAATTAATAGACTGGATGGAGGCGG
ATAAAGTTGCAGGACCACTTCTGCGCTCGGCCCTTCCGGCTGGCTGGTTTATTGCTGATAAATCTGGAGCCGGTGAGCGTGGGTCTCGCGGTATCATTGCAGCACTGGGGCCAGATGGTAAGCCCTCCCGTATCGTAGT
TATCTACACGACGGGGAGTCAGGCAACTATGGATGAACGAAATAGACAGATCGCTGAGATAGGTGCCTCACTGATTAAGCATTGGTAACTGTCAGACCAAGTTTACTCATATATACTTTAGATTGATTTAAAACTTCATT
TTTAATTTAAAAGGATCTAGGTGAAGATCCTTTTTGATAATCTCATGACCAAAATCCCTTAACGTGAGTTTTCGTTCCACTGAGCGTCAGACCCCGTAGAAAAGATCAAAGGATCTTCTTGAGATCCTTTTTTTCTGCGCGT
AATCTGCTGCTTGCAAACAAAAAAACCACCGCTACCAGCGGTGGTTTGTTTGCCGGATCAAGAGCTACCAACTCTTTTTCCGAAGGTAACTGGCTTCAGCAGAGCGCAGATACCAAATACTGTCCTTCTAGTGTAGCCGT
AGTTAGGCCACCACTTCAAGAACTCTGTAGCACCGCCTACATACCTCGCTCTGCTAATCCTGTTACCAGTGGCTGCTGCCAGTGGCGATAAGTCGTGTCTTACCGGGTTGGACTCAAGACGATAGTTACCGGATAAGGCG
CAGCGGTCGGGCTGAACGGGGGGTTCGTGCACACAGCCCAGCTTGGAGCGAACGACCTACACCGAACTGAGATACCTACAGCGTGAGCATTGAGAAAGCGCCACGCTTCCCGAAGGGAGAAAGGCGGACAGGTATC
CGGTAAGCGGCAGGGTCGGAACAGGAGAGCGCACGAGGGAGCTTCCAGGGGGAAACGCCTGGTATCTTTATAGTCCTGTCGGGTTTCGCCACCTCTGACTTGAGCGTCGATTTTTGTGATGCTCGTCAGGGGGGCGG
AGCCTATGGAAAAACGCCAGCAACGCGGCCTTTTTACGGTTCCTGGCCTTTTGCTGGCCTTTTGCTCACATGTTCTTTCCTGCGTTATCCCCTGATTCTGTGGATAACCGTATTACCGCCTTTGAGTGAGCTGATACCGCTC
GCCGCAGCCGAACGACCGAGCGCAGCGAGTCAGTGAGCGAGGAAGCAGAATGCGGCCGCAAGCTTATTTTTTGTATACTGTTTTGTGATAGCACGAAGTTTTTCCACGGTATCTTGTAAAAATATATATTTGTGGCGGG
CTTACCTACATCAAATTAATAAGAGACTAATTATAAACTAAACACACAAGCAAGCTACTTTAGGGTAAAAGTTTATAAATGCTTTTGACGTATAAACGTTGCTTGTATTTATTATTACAATTAAAGGTGGATAGAAAACCT
AGAGACTAGTTAGAAACTAATCTCAGGTTTGCGTTAAACTAAATCAGAGCCCGAGAGGTTAACAGAACCTAGAAGGGGACTAGATATCCGGGTAGGGAAACAAAAAAAAAAACAAGACAGCCACATATTAGGGAGA
CTAGTTAGAAGCTAGTTCCAGGACTAGGAAAATAAAAGACAATGATACCACAGTCTAGTTGACAACTAGATAGATTCTAGATTGAGGCCAAAGTCTCTGAGATCCAGGTTAGTTGCAACTAATACTAGTTAGTATCTAG
TCTCCTATAACTCTGAAGCTAGAATAACTTACTACTATTATCCTCACCACTGTTCAGCTGCGCAAACGGAGTGATTGCAAGGTGTTCAGAGACTAGTTATTGACTAGTCAGTGACTAGCAATAACTAACAAGGTATTAACC
TACCATGTCTGCCATCACCCTGCACTTCCTCGGGCTCAGCAGCCTTTTCCTCCTCATTTTCATGCTCATTTTCCTTGTTTAAGACTGTGACTAGTCAAAGACTAGTCCAGAACCACAAAGGAGAAATGTCTTACCACTTTCTT
CATTGCTTGTCTCTTTTGCATTATCCATGTCTGCAACTAGTTAGAGTCTAGTTAGTGACTAGTCCGACGAGGACTTGCTTGTCTCCGGATTGTTGGAGGAACTCTCCAGGGCCTCAAGATCCACAACAGAGCCTTCTAGAA
GACTGGTCAATAACTAGTTGGTCTTTGTCTGAGTCTGACTTACGAGGTTGCATACTCGCTCCCTTTGCCTCGTCAATCGATGAGAAAAAGCGCCAAAACTCGCAATATGGCTTTGAACCACACGGTGCTGAGACTAGTTA
GAATCTAGTCCCAAACTAGCTTGGATAGCTTACCTTTGCCCTTTGCGTTGCGACAGGTCTTGCAGGGTATGGTTCCTTTCTCACCAGCTGATTTAGCTGCCTTGCTACCCTCACGGCGGATCTGCCATAAAGAGTGGCTAG
AGGTTATAAATTAGCACTGATCCTAGGTACGGGGCTGAATGTAACTTGCCTTTCCTTTCTCATCGCGCGGCAAGACAGGCTTGCTCAAATTCCTACCAGTCACAGGGGTATGCACGGCGTACGGACCACTTGAACTAGTC
ACAGATTAGTTAGCAACTAGTCTGCATTGAAT

Sequence of ANEp8_Cas9_glaA

AMA1, pyrG, gRNAglaA, pKi promoter, Cas9, NLS, GlaA terminator

GGCTGTACTTACGGGCCCTCGCCATTGTCCTGATCATTTCCAGCTTCACCCTCGTTGCTGCAAAGTAGTTAGTGACTAGTCAAGGACTAGTTGAAATGGGAGAAGAAACTCACGAATTCTCGACTCCCTTAGTATTGT
GGTCCTTGGACTTGGTGCTGCTATATATTAGCTAATACACTAGTTAGACTCACAGAAACTTACGCAGCTCGCTTGCGCTTCTTGGTAGGAGTCGGGGTTGGGAGAACAGTGCCTTCAAACAAGCCTTCATACCATGCT
ACTTGACTAGTCAGGGACTAGTCACCAAGTAATCTAGATAGGACTTGCCTTTGGCCTCCATCAGTTCCTTCATAGTGGGAGGACCATTGTGCAATGTAAACTCCATGCCGTGGGAGTTCTTGTCCTTCAAGTGCTTGA
CCAATATGTTTCTGTTGGCAGAGGGAACCTGTCAACTAGTTAATAACTAGTCAGAAACTATGATAGCAGTAGACTCACTGTACGCTTGAGGCATCCCTTCACTCGGCAGTAGACTTCATATGGATGGATATCAGGCA
CGCCATTGTCGTCCTGTGGACTAGTCAGTAACTAGGCTTAAAGCTAGTCGGGTCGGCTTACTATCTTGAAATCCGGCAGCGTAAGCTCCCCGTCCTTAACTGCCTCGAGATAGTGACAGTACTCTGGGGACTTTCGGA
GATCGTTATCGTTATCGCGAATGCTCGGCATACTAACTGTTGACTAGTCTTGGACTAGTCCCGAGCAAAAAGGATTGGAGGAGGAGGAGGAAGGTGAGAGTGAGACAAAGAGCGAAATAAGAGCTTCAAAGGCT
ATCTCTAAGCAGTATGAAGGTTAAGTATCTAGTTCTTGACTAGATTTAAAGAGATTTCGACTAGTTATGTACCTGGAGTTTGGATATAGGAATGTGTTGTGGTAACGAAATGTAAGGGGGAGGAAAGAAAAAGTC
GTCAAGAGGTAACTCTAAGTCGGCCATTCCTTTTTGGGAGGCGCTAACCATAAACGGCATGGTCGACTTAGAGTTAGCTCAGGGAATTTAGGGAGTTATCTGCGACCACTGGACTCTCACCGTTTCCATTGGGGTAC
TAACATAGCCATCAAATGCCGGCCCGTGTTTAATCTCCTCGAGATGGCTGGGCTCACATGCGTTCTACTCAAAAACTCTTATACCTAGGCCCTCTCCCAATCGTGTATGTTTGGACCTTCCTCTCTCACACTTTGGATAA
CATCAAGCATACTGCAGATTGGTACCTAGTTAACAAGGCACATCATGCTATCACTTCCAACACTCTTGCTGAGCGCGTTCTGCCTTGCCTTTCTGGTTCTACGAAGTATCCAGTCCCGCCTGCAACGCCGCAGAAACGC
CAAAAGGTGGGGTTGTCAACCGCCAGCTAGAGCTCCATCGGGATTCTTTGGCATTCCGCCGTTCCTCGGTGGTCGCAGATAACTCCCTAAATTCCCTGAGCTAACTCTAAGTCGACCATGCCGTTTATGGTTAGCGCC
TCCCAAAAAGGAATGGCCGACTTAGAGTTACCTCTTGACGACTTTTTCTTTCCTCCCCCTTACATTTCGTTACCACAACACATTCCTATATCCAAACTCCAGGTACATAACTAGTCGAAATCTCTTTAAATCTAGTCAAG
AACTAGATACTTAACCTTCATACTGCTTAGAGATAGCCTTTGAAGCTCTTATTTCGCTCTTTGTCTCACTCTCACCTTCCTCCTCCTCCTCCAATCCTTTTTGCTCGGGACTAGTCCAAGACTAGTCAACAGTTAGTATGCC
GAGCATTCGCGATAACGATAACGATCTCCGAAAGTCCCCAGAGTACTGTCACTATCTCGAGGCAGTTAAGGACGGGGAGCTTACGCTGCCGGATTTCAAGATAGTAAGCCGACCCGACTAGCTTTAAGCCTAGTTA
CTGACTAGTCCACAGGACGACAATGGCGTGCCTGATATCCATCCATATGAAGTCTACTGCCGAGTGAAGGGATGCCTCAAGCGTACAGTGAGTCTACTGCTATCATAGTTTCTGACTAGTTATTAACTAGTTGACAG
GTTCCCTCTGCCAACAGAAACATATTGGTCAAGCACTTGAAGGACAAGAACTCCCACGGCATGGAGTTTACATTGCACAATGGTCCTCCCACTATGAAGGAACTGATGGAGGCCAAAGGCAAGTCCTATCTAGATT
ACTTGGTGACTAGTCCCTGACTAGTCAAGTAGCATGGTATGAAGGCTTGTTTGAAGGCACTGTTCTCCCAACCCCGACTCCTACCAAGAAGCGCAAGCGAGCTGCGTAAGTTTCTGTGAGTCTAACTAGTGTATTAG
CTAATATATAGCAGCACCAAGTCCAAGGACCACAATACTAAGGGAGTCGAGAATTCGTGAGTTTCTTCTCCCATTTCAACTAGTCCTTGACTAGTCACTAACTACTTTGCAGCAACGAGGGTGAAGCTGGAAATGAT
CAGGACAATGGCGAGGGCCCGTAAGTACAGCCATTCAATGCAGACTAGTTGCTAACTAATCTGTGACTAGTTCAAGTGGTCCGTACGCCGTGCATACCCCTGTGACTGGTAGGAATTTGAGCAAGCCTGTCTTGCCG
CGCGATGAGAAAGGAAAGGCAAGTTACATTCAGCCCCGTACCTAGGATCAGTGCTAATTTATAACCTCTAGCCACTCTTTATGGCAGATCCGCCGTGAGGGTAGCAAGGCAGCTAAATCAGCTGGTGAGAAAGGA
ACCATACCCTGCAAGACCTGTCGCAACGCAAAGGGCAAAGGTAAGCTATCCAAGCTAGTTTGGGACTAGATTCTAACTAGTCTCAGCACCGTGTGGTTCAAAGCCATATTGCGAGTTTTGGCGCTTTTTCTCATCGAT
TGACGAGGCAAAGGGAGCGAGTATGCAACCTCGTAAGTCAGACTCAGACAAAGACCAACTAGTTATTGACCAGTCTTCTAGAAGGCTCTGTTGTGGATCTTGAGGCCCTGGAGAGTTCCTCCAACAATCCGGAGAC
AAGCAAGTCCTCGTCGGACTAGTCACTAACTAGACTCTAACTAGTTGCAGACATGGATAATGCAAAAGAGACAAGCAATGAAGAAAGTGGTAAGACATTTCTCCTTTGTGGTTCTGGACTAGTCTTTGACTAGTCAC
AGTCTTAAACAAGGAAAATGAGCATGAAAATGAGGAGGAAAAGGCTGCTGAGCCCGAGGAAGTGCAGGGTGATGGCAGACATGGTAGGTTAATACCTTGTTAGTTATTGCTAGTCACTGACTAGTCAATAACTA
GTCTCTGAACACCTTGCAATCACTCCGTTTGCGCAGCTGAACAGTGGTGAGGATAATAGTAGTAAGTTATTCTAGCTTCAGAGTTATAGGAGACTAGATACTAACTAGTATTAGTTGCAACTAACCTGGATCTCAGA
GACTTTGGCCTCAATCTAGAATCTATCTAGTTGTCAACTAGACTGTGGTATCATTGTCTTTTATTTTCCTAGTCCTGGAACTAGCTTCTAACTAGTCTCCCTAATATGTGGCTGTCTTGTTTTTTTTTTTTTGTTTCCCTACCC
GGATATCTAGTCCCCTTCTAGGTTCTGTTAACCTCTCGGGCTCTGATTTAGTTTAACGCAAACCTGAGATTAGTTTCTAACTAGTCTCTAGGTTTTCTATCCACCTTTAATTGTAATAATAAATACAAGCAACGTTTATA
CGTCAAAAGCATTTATAAACTTTTACCCTAAAGTAGCTTGCTTGTGTGTTTAGTTTATAATTAGTCTCTTATTAATTTGATGTAGGTAAGCCCGCCACAAATATATATTTTTACAAGATACCGTGGAAAAACTTCGTGCT
ATCACAAAACAGTATACAAAAAATAAGCTTGCGGCCGCGCAACTTCCTCGAGAACGCGCCGCAGACAATGCTCTCTATCCTGGTGGCAGGCGTCAAGTACCCAGAGGCAGCAGCGGGCTTAGGAGCGGCCTGGGTTG
TTCTCCGCACCCTCTACATGCTGGGCTATATTTATAGCGACAAGCCGAACGGCACCGGCAGGTACAATGGTTCGCTGTACTTGCTTGCGCAAGCGGGTCTTTGGGGATTGAGCGCATTTGGTGTTGCAAAGGATTTGAT
GTAAATGTAGTCGACATCTTAGCACAGAGGGGAGAGTTGATAAAATGTGGTCTGTTTGAATGATAGTCGGGTTCGTGACCTATATTCGTGATAGTGGAGATAGGTCTGCGCCTATCTTATCGGGCCGGAGCAAAAATTC
CACCGCAGCGGGGTGAGTTTTCGTTATACAGCCATCCCACTTCCAGCTTCAAATTGTCAGTTTAATCCAGCCCAATTCAATCATTGGAGAACCGCCATCATGTCTTCGAAGTCCCACCTCCCCTACGCAATTCGCGCAACC
AACCATCCCAACCCTTTAACATCTAAACTCTTCTCCATCGCCGAGGAGAAGAAAACCAACGTCACCGTCTCCGCAGACGTTACTACTTCCGCCGAGCTCCTCGATCTTGCTGACCGTACATCCTGCACCAATGCCCCTCCA
GGATAACAAATAGCTGATGCGTAGTGAGTACAGGCCTAGGCCCCTATATCGCAGTTCTGAAAACCCACATCGACATCCTCACCGATCTCACCCCGTCGACCCTTTCCTCGCTCCAATCCCTCGCGACAAAGCACAACTTCC
TCATCTTTGAGGACCGCAAGTTCATCGACATCGGCAACACCGTGCAAAAGCAGTACCACGGTGGCGCTCTCCGCATCTCCGAATGGGCACACATCATCAACTGCGCCATCCTGCCGGGCGAAGGGATCGTCGAGGCCCT
CGCACAGACAACCAAGTCTCCTGACTTTAAAGACGCGAATCAACGAGGTCTCCTGATTCTTGCCGAGATGACGAGTAAGGGATCTCTTGCGACAGGGGAGTCACAGGCACGCTCGGTTGAGTACGCGCGGAAGTATAA
GGGGTTTGTGATGGGATTCGTGAGTACAAGGGCGTTGAGTGAGGTGCTGCCCGAACAGAAAGAGGAGAGCGAGGATTTTGTCGTCTTTACGACTGGGGTGAATCTGTCGGATAAGGGGGATAAGCTGGGGCAGCAG
TATCAGACACCTGGGTCGGCGGTTGGGCGAGGTGCGGACTTTATCATTGCGGGTAGGGGCATCTATAAGGCGGACGATCCAGTCGAGGCGGTTCAGAGGTACCGGGAGGAAGGCTGGAAAGCTTACGAGAAAAGA
GTTGGACTTTGAGTGTGAGTGGAAATGTGTAACGGTATTGACTAAAAGGGGACGTCCCACATCTGATGCCATTGGCGGAGGGGTCCGGACGGTCAGGAACTTAGCCTTATGAGATGAATGATGGACGTGTCTGGCCT
CGGAAAAGGATATATGGGGATCATAATAGTACTAGCCATATTAATGAAGGGCATATACCACGCGACTACTCTACCACTATTTGAAAAGCAAAAAAGGAAGGTACAAAAAAGCACCGACTCGGTGCCACTTTTTCAAGTT
GATAACGGACTAGCCTTATTTTAACTTGCTATTTCTAGCTCTAAAACCGAGGTGACAGTCACACTGCGACGAGCTTACTCGTTTCGTCCTCACGGACTCATCAGAGTGGGGGCCCGAGCCGCGGATCGAACGCGGGACC
TCTCGCAGATATGCTTGGGTTTGTATCCCTAAGCGAGAATCATACCACTAGACCACCCGGGCTTGTTATGGTAACTGCCTCTAACTTTGGCTTTATTAGCGGCCAGTAAATATCTATCATAACAGCAAGTTGCAAATGACC
TACCAGTACGTTCGGCGGAGTCAAATTGGATTGGAGGTTGCGGCGCGCCGTTTAAACAAAGCAGCACGCGTAAATGGAAGAGAAAACCTCCGAGTACTTACTTAGGGCCCTGTCTACTGGCCAGAGTCTCGTCCTCTAT
TCACTATGATAAATTACCCACTGGACAAAAAAATAAAATAAAATAAAAATAAAAAGGGAGACAGCTTCTCCATCAACTGGCAACTGGGTCCGTCCGAGCAGAGCAAAATTCAGCCTTATGGGTTCCGATGGAGTCAGG
GAAATAGTTCTTGCGAAGGGCATTGGGCTTTTTTGCGAGGAGAAAATTCAGCACCGACAAAGCATCCGAAATCCGACCTCGCTAGGAGAGAATGGATCCGCGACGATGTGGGGTCAACTGGACAGAGTGAGAGGGTA
TCATGTGGTCCTGCCAGATACTTCGCAGAATGTTGTGTGGGTGTCTGATTGTGGCTTGGGCGTGAATTGCTTTTGGTCTTCCCAACCAATTATTATTGCATGCGGCGTATGAATGCCTGAGATGCGCGGAGGGAAGGTG
CCTGAGGATGTAGTGGACAAATGCTGCTGATCGCTGGGCGGAAACCCTTGGCTGACCAGTGAAAAGAGCGGACGGAGGCAGCAGGTGTATCTACGATCAAAGAATAGTAGCAAAGCAGTGAAAGGTGGATCACCCA

GCAAATAATTGAGTTTTGATACCCAGCGATAGTGCCGGGGGGGAGAAAAAGTCATTAATAATGGGAATTATGTAGGCGATGGGAAGTGTGATTGTAACTACTCCGTAGCTGGAGGCACAACTAACAAGCCAGCTCTCA
ACCCGCGGGGAACCGACCGACAGATAAAAAAAAAGCGTCCCAAAGCAGGAATCCCACCAAAAAGGGCCGATCCAGCCAATCACCGCCGCCAACATTTTTCCTTCCCGGGCACCCCTCCTCTAGTCCACCATCTCTCTCTTC
TCTCGCTCACCGGCCCCGTCTTTTCCTTCCCTATTATCTCTCCCTCTTCTCCTCCCTTCTCTCCCTCCATTCTTTCTCCCATCTTCATCACTCCCTTCTCTTCTGTCTTCCCCCCCGGTTCAGTAGAGATCAATCAGCTAGCTTAAT
TAAATGGACAAGAAGTACTCCATTGGGCTCGCTATCGGCACAAACAGCGTCGGCTGGGCCGTCATTACGGACGAGTACAAGGTGCCGAGCAAAAAATTCAAAGTTCTGGGCAATACCGATCGCCACAGCATAAAGAA
GAACCTCATTGGCGCCCTCCTGTTCGACTCCGGGGAGACGGCCGAAGCCACGCGGCTCAAAAGAACAGCACGGCGCAGATATACCCGCAGAAAGAATCGGATCTGCTACCTGCAGGAGATCTTTAGTAATGAGATGG
CTAAGGTGGATGACTCTTTCTTCCATAGGCTGGAGGAGTCCTTTTTGGTGGAGGAGGATAAAAAGCACGAGCGCCACCCAATCTTTGGCAATATCGTGGACGAGGTGGCGTACCATGAAAAGTACCCAACCATATATCA
TCTGAGGAAGAAGCTTGTAGACAGTACTGATAAGGCTGACTTGCGGTTGATCTATCTCGCGCTGGCGCATATGATCAAATTTCGGGGACACTTCCTCATCGAGGGGGACCTGAACCCAGACAACAGCGATGTCGACAA
ACTCTTTATCCAACTGGTTCAGACTTACAATCAGCTTTTCGAAGAGAACCCGATCAACGCATCCGGAGTTGACGCCAAAGCAATCCTGAGCGCTAGGCTGTCCAAATCCCGGCGGCTCGAAAACCTCATCGCACAGCTCC
CTGGGGAGAAGAAGAACGGCCTGTTTGGTAATCTTATCGCCCTGTCACTCGGGCTGACCCCCAACTTTAAATCTAACTTCGACCTGGCCGAAGATGCCAAGCTTCAACTGAGCAAAGACACCTACGATGATGATCTCGA
CAATCTGCTGGCCCAGATCGGCGACCAGTACGCAGACCTTTTTTTGGCGGCAAAGAACCTGTCAGACGCCATTCTGCTGAGTGATATTCTGCGAGTGAACACGGAGATCACCAAAGCTCCGCTGAGCGCTAGTATGATC
AAGCGCTATGATGAGCACCACCAAGACTTGACTTTGCTGAAGGCCCTTGTCAGACAGCAACTGCCTGAGAAGTACAAGGAAATTTTCTTCGATCAGTCTAAAAATGGCTACGCCGGATACATTGACGGCGGAGCAAGC
CAGGAGGAATTTTACAAATTTATTAAGCCCATCTTGGAAAAAATGGACGGCACCGAGGAGCTGCTGGTAAAGCTTAACAGAGAAGATCTGTTGCGCAAACAGCGCACTTTCGACAATGGAAGCATCCCCCACCAGATTC
ACCTGGGCGAACTGCACGCTATCCTCAGGCGGCAAGAGGATTTCTACCCCTTTTTGAAAGATAACAGGGAAAAGATTGAGAAAATCCTCACATTTCGGATACCCTACTATGTAGGCCCCCTCGCCCGGGGAAATTCCAG
ATTCGCGTGGATGACTCGCAAATCAGAAGAGACCATCACTCCCTGGAACTTCGAGGAAGTCGTGGATAAGGGGGCCTCTGCCCAGTCCTTCATCGAAAGGATGACTAACTTTGATAAAAATCTGCCTAACGAAAAGGT
GCTTCCTAAACACTCTCTGCTGTACGAGTACTTCACAGTTTATAACGAGCTCACCAAGGTCAAATACGTCACAGAAGGGATGAGAAAGCCAGCATTCCTGTCTGGAGAGCAGAAGAAAGCTATCGTGGACCTCCTCTTC
AAGACGAACCGGAAAGTTACCGTGAAACAGCTCAAAGAAGACTATTTCAAAAAGATTGAATGTTTCGACTCTGTTGAAATCAGCGGAGTGGAGGATCGCTTCAACGCATCCCTGGGAACGTATCACGATCTCCTGAAA
ATCATTAAAGACAAGGACTTCCTGGACAATGAGGAGAACGAGGACATTCTTGAGGACATTGTCCTCACCCTTACGTTGTTTGAAGATAGGGAGATGATTGAAGAACGCTTGAAAACTTACGCTCATCTCTTCGACGACA
AAGTCATGAAACAGCTCAAGAGGCGCCGATATACAGGATGGGGGCGGCTGTCAAGAAAACTGATCAATGGGATCCGAGACAAGCAGAGTGGAAAGACAATCCTGGATTTTCTTAAGTCCGATGGATTTGCCAACCGG
AACTTCATGCAGTTGATCCATGATGACTCTCTCACCTTTAAGGAGGACATCCAGAAAGCACAAGTTTCTGGCCAGGGGGACAGTCTTCACGAGCACATCGCTAATCTTGCAGGTAGCCCAGCTATCAAAAAGGGAATAC
TGCAGACCGTTAAGGTCGTGGATGAACTCGTCAAAGTAATGGGAAGGCATAAGCCCGAGAATATCGTTATCGAGATGGCCCGAGAGAACCAAACTACCCAGAAGGGACAGAAGAACAGTAGGGAAAGGATGAAGAG
GATTGAAGAGGGTATAAAAGAACTGGGGTCCCAAATCCTTAAGGAACACCCAGTTGAAAACACCCAGCTTCAGAATGAGAAGCTCTACCTGTACTACCTGCAGAACGGCAGGGACATGTACGTGGATCAGGAACTGG
ACATCAATCGGCTCTCCGACTACGACGTGGATCATATCGTGCCCCAGTCTTTTCTCAAAGATGATTCTATTGATAATAAAGTGTTGACAAGATCCGATAAAAATAGAGGGAAGAGTGATAACGTCCCCTCAGAAGAAGTT
GTCAAGAAAATGAAAAATTATTGGCGGCAGCTGCTGAACGCCAAACTGATCACACAACGGAAGTTCGATAATCTGACTAAGGCTGAACGAGGTGGCCTGTCTGAGTTGGATAAAGCCGGCTTCATCAAAAGGCAGCTT
GTTGAGACACGCCAGATCACCAAGCACGTGGCCCAAATTCTCGATTCACGCATGAACACCAAGTACGATGAAAATGACAAACTGATTCGAGAGGTGAAAGTTATTACTCTGAAGTCTAAGCTGGTCTCAGATTTCAGAA
AGGACTTTCAGTTTTATAAGGTGAGAGAGATCAACAATTACCACCATGCGCATGATGCCTACCTGAATGCAGTGGTAGGCACTGCACTTATCAAAAAATATCCCAAGCTTGAATCTGAATTTGTTTACGGAGACTATAAA
GTGTACGATGTTAGGAAAATGATCGCAAAGTCTGAGCAGGAAATAGGCAAGGCCACCGCTAAGTACTTCTTTTACAGCAATATTATGAATTTTTTCAAGACCGAGATTACACTGGCCAATGGAGAGATTCGGAAGCGAC
CACTTATCGAAACAAACGGAGAAACAGGAGAAATCGTGTGGGACAAGGGTAGGGATTTCGCGACAGTCCGGAAGGTCCTGTCCATGCCGCAGGTGAACATCGTTAAAAAGACCGAAGTACAGACCGGAGGCTTCTCC
AAGGAAAGTATCCTCCCGAAAAGGAACAGCGACAAGCTGATCGCACGCAAAAAAGATTGGGACCCCAAGAAATACGGCGGATTCGATTCTCCTACAGTCGCTTACAGTGTACTGGTTGTGGCCAAAGTGGAGAAAGG
GAAGTCTAAAAAACTCAAAAGCGTCAAGGAACTGCTGGGCATCACAATCATGGAGCGATCAAGCTTCGAAAAAAACCCCATCGACTTTCTCGAGGCGAAAGGATATAAAGAGGTCAAAAAAGACCTCATCATTAAGCT
TCCCAAGTACTCTCTCTTTGAGCTTGAAAACGGCCGGAAACGAATGCTCGCTAGTGCGGGCGAGCTGCAGAAAGGTAACGAGCTGGCACTGCCCTCTAAATACGTTAATTTCTTGTATCTGGCCAGCCACTATGAAAAG
CTCAAAGGGTCTCCCGAAGATAATGAGCAGAAGCAGCTGTTCGTGGAACAACACAAACACTACCTTGATGAGATCATCGAGCAAATAAGCGAATTCTCCAAAAGAGTGATCCTCGCCGACGCTAACCTCGATAAGGTG
CTTTCTGCTTACAATAAGCACAGGGATAAGCCCATCAGGGAGCAGGCAGAAAACATTATCCACTTGTTTACTCTGACCAACTTGGGCGCGCCTGCAGCCTTCAAGTACTTCGACACCACCATAGACAGAAAGCGGTACA
CCTCTACAAAGGAGGTCCTGGACGCCACACTGATTCATCAGTCAATTACGGGGCTCTATGAAACAAGAATCGACCTCTCTCAGCTCGGTGGAGACAGCAGGGCTGACCCCAAGAAGAAGAGGAAGGTGTAAGGCCGG
CCCGACCGCGACGGTGACTGACACCTGGCGGTAGACAATCAATCCATTTCGCTATAGTTAAAGGATGGGGATGAGGGCAATTGGTTATATGATCATGTATGTAGTGGGTGTGCATAATAGTAGTGAAATGGAAGCCAA
GTCATGTGATTGTAATCGACCGACGGAATTGAGGATATCCGGAAATACAGACACCGTGAAAGCCATGGTCTTTCCTTCGTGTAGAAGACCAGACAGACAGTCCCTGATTTACCCTGCACAAAGCACTAGAAAATTAGCA
TTCCATCCTTCTCTGCTTGCTCTGCTGATATCACTGTCATTCAATGCATAGCCATGAGCTCATCTTAGATCCAAGCACGTAATTCCATAGCCGAGGTCCACAGTGGAGCAGCAACATTCCCCATCATTGCTTTCCCCAGGGG
CCTCCCAACGACTAAATCAAGAGTATATCTCTACCGTCCAATAGATCGTCTTCGCTTCAAAATCTTTGACAATTCCAAGAGGGTCCCCATCCATCAAACCCAGTTCAATAATAGCCGAGATGCATGGTGGAGTCAATTAGG
CAGTATTGCTGGAATGTCGGGGCCAGTTCCGGGTGGTCATTGGCCGCCTGTGATGCCATCTGCCACTAAATCCGATCATTGATCCACCGCCCACGAGGGCGTCTTTGCTTTTTGCGCGGCGTCCAGGTTCAACTCTCTCT
GCAGCTCCAGTCCAACGCTGACTGACTAGTTTACCTACTGGTCTGATCGGCTCCATCAGAGCTATGGCGTTATCCCGTGCCGTTGCTGCGCAATCGCTATCTTGATCGCAACCTTGAACTCACTCTTGTTTTAATAGTGATC
TTGGTGACGGAGTGTCGGTGAGTGACAACCAACATCGTGCAAGGGAGATTGATACGGAATTGTCGCTCCCATCATGATGTTCTTGCCGGCTTTGTTGGCCCTATTCGTGGGATCGATGCCCTCCTGTGCAGCAGCAGGT
ACTGCTGGATGAGGAGCCATCGGTCTCTGCACGCAAACCCAACTTCCTCTTCATTCTCACGGATGATCAGGATCTCCGGAATATTCGCGGACCTGCAGGCATGCAAGCTTGGCACTGGCCGTCGTTTTACAACGTCGTGA
CTGGGAAAACCCTGGCGTTACCCAACTTAATCGCCTTGCAGCACATCCCCCTTTCGCCAGCTGGCGTAATAGCGAAGAGGCCCGCACCGATCGCCCTTCCCAACAGTTGCGCAGCCTGAATGGCGAATGGCGCCTGATG
CGGTATTTTCTCCTTACGCATCTGTGCGGTATTTCACACCGCATATGGTGCACTCTCAGTACAATCTGCTCTGATGCCGCATAGTTAAGCCAGCCCCGACACCCGCCAACACCCGCTGACGCGCCCTGACGGGCTTGTCTG
CTCCCGGCATCCGCTTACAGACAAGCTGTGACCGTCTCCGGGAGCTGCATGTGTCAGAGGTTTTCACCGTCATCACCGAAACGCGCGAGACGAAAGGGCCTCGTGATACGCCTATTTTTATAGGTTAATGTCATGATAAT
AATGGTTTCTTAGCAGGTGGCACTTTTCGGGGAAATGTGCGCGGAACCCCTATTTGTTTATTTTTCTAAATACATTCAAATATGTATCCGCTCATGAGACAATAACCCTGATAAATGCTTCAATAATATTGAAAAAGGAAG
AGTATGAGTATTCAACATTTCCGTGTCGCCCTTATTCCCTTTTTTGCGGCATTTTGCCTTCCTGTTTTTGCTCACCCAGAAACGCTGGTGAAAGTAAAAGATGCTGAAGATCAGTTGGGTGCACGAGTGGGTTACATCGAA
CTGGATCTCAACAGCGGTAAGATCCTTGAGAGTTTTCGCCCCGAAGAACGTTTTCCAATGATGAGCACTTTTAAAGTTCTGCTATGTGGCGCGGTATTATCCCGTATTGACGCCGGGCAAGAGCAACTCGGTCGCCGCAT
ACACTATTCTCAGAATGACTTGGTTGAGTACTCACCAGTCACAGAAAAGCATCTTACGGATGGCATGACAGTAAGAGAATTATGCAGTGCTGCCATAACCATGAGTGATAACACTGCGGCCAACTTACTTCTGACAACG
ATCGGAGGACCGAAGGAGCTAACCGCTTTTTTGCACAACATGGGGGATCATGTAACTCGCCTTGATCGTTGGGAACCGGAGCTGAATGAAGCCATACCAAACGACGAGCGTGACACCACGATGCCTGTAGCAATGGCA
ACAACGTTGCGCAAACTATTAACTGGCGAACTACTTACTCTAGCTTCCCGGCAACAATTAATAGACTGGATGGAGGCGGATAAAGTTGCAGGACCACTTCTGCGCTCGGCCCTTCCGGCTGGCTGGTTTATTGCTGATAA
ATCTGGAGCCGGTGAGCGTGGGTCTCGCGGTATCATTGCAGCACTGGGGCCAGATGGTAAGCCCTCCCGTATCGTAGTTATCTACACGACGGGGAGTCAGGCAACTATGGATGAACGAAATAGACAGATCGCTGAGA
TAGGTGCCTCACTGATTAAGCATTGGTAACTGTCAGACCAAGTTTACTCATATATACTTTAGATTGATTTAAAACTTCATTTTTAATTTAAAAGGATCTAGGTGAAGATCCTTTTTGATAATCTCATGACCAAAATCCCTTAA
CGTGAGTTTTCGTTCCACTGAGCGTCAGACCCCGTAGAAAAGATCAAAGGATCTTCTTGAGATCCTTTTTTTCTGCGCGTAATCTGCTGCTTGCAAACAAAAAAACCACCGCTACCAGCGGTGGTTTGTTTGCCGGATCAA
GAGCTACCAACTCTTTTTCCGAAGGTAACTGGCTTCAGCAGAGCGCAGATACCAAATACTGTCCTTCTAGTGTAGCCGTAGTTAGGCCACCACTTCAAGAACTCTGTAGCACCGCCTACATACCTCGCTCTGCTAATCCTG
TTACCAGTGGCTGCTGCCAGTGGCGATAAGTCGTGTCTTACCGGGTTGGACTCAAGACGATAGTTACCGGATAAGGCGCAGCGGTCGGGCTGAACGGGGGGTTCGTGCACACAGCCCAGCTTGGAGCGAACGACCTA
CACCGAACTGAGATACCTACAGCGTGAGCATTGAGAAAGCGCCACGCTTCCCGAAGGGAGAAAGGCGGACAGGTATCCGGTAAGCGGCAGGGTCGGAACAGGAGAGCGCACGAGGGAGCTTCCAGGGGGAAACGC
CTGGTATCTTTATAGTCCTGTCGGGTTTCGCCACCTCTGACTTGAGCGTCGATTTTTGTGATGCTCGTCAGGGGGGCGGAGCCTATGGAAAAACGCCAGCAACGCGGCCTTTTTACGGTTCCTGGCCTTTTGCTGGCCTTT
TGCTCACATGTTCTTTCCTGCGTTATCCCCTGATTCTGTGGATAACCGTATTACCGCCTTTGAGTGAGCTGATACCGCTCGCCGCAGCCGAACGACCGAGCGCAGCGAGTCAGTGAGCGAGGAAGCAGAATGCGGCCGC
AAGCTTATTTTTTGTATACTGTTTTGTGATAGCACGAAGTTTTTCCACGGTATCTTGTAAAAATATATATTTGTGGCGGGCTTACCTACATCAAATTAATAAGAGACTAATTATAAACTAAACACACAAGCAAGCTACTTTA
GGGTAAAAGTTTATAAATGCTTTTGACGTATAAACGTTGCTTGTATTTATTATTACAATTAAAGGTGGATAGAAAACCTAGAGACTAGTTAGAAACTAATCTCAGGTTTGCGTTAAACTAAATCAGAGCCCGAGAGGTTA
ACAGAACCTAGAAGGGGACTAGATATCCGGGTAGGGAAACAAAAAAAAAAAACAAGACAGCCACATATTAGGGAGACTAGTTAGAAGCTAGTTCCAGGACTAGGAAAATAAAAGACAATGATACCACAGTCTAGTTG
ACAACTAGATAGATTCTAGATTGAGGCCAAAGTCTCTGAGATCCAGGTTAGTTGCAACTAATACTAGTTAGTATCTAGTCTCCTATAACTCTGAAGCTAGAATAACTTACTACTATTATCCTCACCACTGTTCAGCTGCGCA
AACGGAGTGATTGCAAGGTGTTCAGAGACTAGTTATTGACTAGTCAGTGACTAGCAATAACTAACAAGGTATTAACCTACCATGTCTGCCATCACCCTGCACTTCCTCGGGCTCAGCAGCCTTTTCCTCCTCATTTTCATG
CTCATTTTCCTTGTTTAAGACTGTGACTAGTCAAAGACTAGTCCAGAACCACAAAGGAGAAATGTCTTACCACTTTCTTCATTGCTTGTCTCTTTTGCATTATCCATGTCTGCAACTAGTTAGAGTCTAGTTAGTGACTAGTC
CGACGAGGACTTGCTTGTCTCCGGATTGTTGGAGGAACTCTCCAGGGCCTCAAGATCCACAACAGAGCCTTCTAGAAGACTGGTCAATAACTAGTTGGTCTTTGTCTGAGTCTGACTTACGAGGTTGCATACTCGCTCCC
TTTGCCTCGTCAATCGATGAGAAAAAGCGCCAAAACTCGCAATATGGCTTTGAACCACACGGTGCTGAGACTAGTTAGAATCTAGTCCCAAACTAGCTTGGATAGCTTACCTTTGCCCTTTGCGTTGCGACAGGTCTTGC
AGGGTATGGTTCCTTTCTCACCAGCTGATTTAGCTGCCTTGCTACCCTCACGGCGGATCTGCCATAAAGAGTGGCTAGAGGTTATAAATTAGCACTGATCCTAGGTACGGGGCTGAATGTAACTTGCCTTTCCTTTCTCAT
CGCGCGGCAAGACAGGCTTGCTCAAATTCCTACCAGTCACAGGGGTATGCACGGCGTACGGACCACTTGAACTAGTCACAGATTAGTTAGCAACTAGTCTGCATTGAAT

Sequence of adaR (NRRL3_09545) cassette

glaA promoter, adaR coding sequence, glaA terminator

GAATGATGGACGTGTCTGGCCTCGGAAAAGGATATATGGGGATCATAATAGTACTAGCCATATTAATGAAGGGCATATACCACGCGTTGGACCTGCGTTATAGCTTCCCGTTAGTTATAGTACCATCGTTATACCA
GCCAATCAAGTCACCACGCACGACCGGGGACGGCGAATCCCCGGGAATTGAAAGAAATTGCATCCCAGGCCAGTGAGGCCAGCGATTGGCCACCTCTCCAAGCACACAGGGCCATTCTGCAGCGCTGGTGGATT
CATCGCAATTTCCCCCGGCCCGGCCCGACACCGCTATAGGCTGGTTCTCCCACACCATCGGAGATTCGTCGCCTAATGTCTCGTCCGTTCACAAGCTGAAGAGCTTGAAGTGGCGAGATGTCTCTGCAGGAATTCA
AGCTAGATGCTAAGCGATATTGCATGGCAATATGTGTTGATGCATGTGCTTCTTCCTTCAGCTTCCCCTCGTGCAGATGAGGTTTGGCTATAAATTGAAGTGGTTGGTCGGGGTTCCGTGAGGGGCTGAAGTGCTT
CCTCCCTTTTAGACGCAACTGAGAGCCTGAGCTTCATCCCCAGCATCATTACACGCTAGATGGAACAACGCAGTTCCCCCGCCAGATCGCTGCCCCCGCGAAAAACCACCACGACTCCCCAGCTCAGTTGCGAACT
CTGCCGCAAGCGCAAGGTCAAGTGCGACAAGCTCACCCCCTGTACCAACTGTGCAGCATCGGGGACGGTCTGTGTCCCCATCTATCGCACACGACTACCCCGAGGCCGACATGCCACTCGTCCGCGTCGAGTGTC
ATCGCCTCCGCCTACATCAGCTCCCGGGGAGACCGATCGGATAATCCAGCCATCTGTCCCAGTCAATGAGGATCTCCAGGAACGAATATACCGACTGGAAGCTCTCATCCAGGGGATGAACTCCCACTCCCATACC
CGAACACCATCCGCCACCTCGCGCGAACAGGTGAGGCTGCACTCTGACCTTTTCAGCCTCGCGGAATGCTGACCCCGACAATGATCATTAGTCCGTCCAATTATCCGACACTTCCACATTCCAAACTGCTCCCAATC
CTAATACTTCCCCCATCCTCAATAGTTCTATTGTTTCCAAGCGTCTCATGTTGCAACGACCCGATCAGTTCTGGGCCGACTTGGTGGATGAAGTAGGTCTGCCAACATGGACCAAGGAAGGAAAGAATGCTGACGC
GGACACATGCAGATTCACGGACTCCGCGAAGTGGTCGAATCATCTCTGGCAGGAGGACAAGAGGGCCCGATCCCTTCCTCCGACTCGGCCAAGTCCGAGCCGCCCAATGATGACGGAATCCAGGTTCTTGGCCT
GGGAGCTAGCAATCCTTCTGCAGCCCTACGGTCCATGAGTCCGTTGCATAATCCGGTGGTCGCCCGTCAGCTCTGTGAAGTGTATCTCCAACAGGTCGATCCAGTGATTAAGATCCTCCATCGTCCGTCGCTGAAC

CGATGGATGGTTCAAGGGGAACCGTATTTATCGTATGCAGATGGTCACCCCGCGGTAGAGGCACTGGGTTCCGCCGTCTGCTACTCGGCTATTAGCAGCATGACGGACAACCAGTGCTCCGTAATGTTTCATGCC
AACAAGGCAGATTTATTGGCCGAGGCTCGTGTAGCCTGTGAGACGGCAATTGGACGTGCGGGTCTGCTCACCACCCGCGATATTACCGTTTTGCAAGCGTTTGTTTTGTATCTGGTATGACTCGACACTGGTCCCA
AGAAGAAAGACGATAAACTGACTCCTTGTAGGTAGCTAGGAGATCTGAAGACCGCACTCCGGCCGTCTGGACACTGATCGCACTGGCTGTGCGAATCGGTAAAGGCCTCGGTCTTTATCTGGATCCCGAGACGG
AGACCTTTTTCGATCAGCAGATACGGAGGCGCCTCTGGTTTACCATTTGCCTGATGGATTTGCAGGCCTCCTTCGGCCAGGCATCTGAGCCGCTGATTAGTGTCGACGAGTCTGCATCCACAGCTCTTCCCCAGCAC
ATCAATGACTCGGACTTTGATCCGACCACGGCCGCCCACAGCGATCCAAACAGGGAGGGTCTGACAGACACGACCTTTGCCTTGGTCACCTACCATGCTCAGCGGACGGGCCGTCTTCTCAACTTTGTTCAACACG
ATCGCAAGGTCGACGGGGGGATTCCAACTCCCACATCCTCTACTTCCGGTACTTCTACATCCCGCTCTCGTACATGCGACCCCAGTTGGCCGCAGCAGCAGGCCCGGCACTTCGAACAAGAAGCACTGCGGCTCCT
CCATTTCTGTGACCCTGGCACCTCTGCGTACGCCTGGTTCACCTGGCACGGCACGCAGAGCCTAATCGCGACAGTCCGACTGGCAGCCGCGCGACCGCTTCAATGGCACGGTCAGGCGCCGCCGCCTCGCCGCGA
GGGCAACACCGAGCTGCTGCGTCTCTGTCTCCCCGTGTTGGAGAAAGCGCAACTCATGCACACCGACCCCCGTGCCGAGGGCTTCCGTTGGTATGTAACAATTCCCTGGTACGCGCTGGCGATGGCCCTCGCAGA
GTGCTATGTGAGCAGTGACACTGCCCTGGTGCGGTACGCCTGGCCGCTGGTCGAGTCGTCGTACCTGCAGTACGAGGCGACGCTGGGACAATCTCTCGGCGGCCCATTCGGACAACTCATGCGGCGCATGAAAG
AGAAGCTGGCGGCGCCGGCCGCACTCCCACCAAGCAGTCTGCCGAGCACCAACTGGAGTCCAGCCACTCCCCCAACATTCCCGGGCGTGCCTCGGCCACAGAGTAGTCATGATGATCGACATGCGCCCGGTTGC
TCTTGGCCGGTGCCCACCGGATCCACCCCGCCTGCGGATCTGGGGGTGCCATCCCTCCTGCCAGTATCCACCTGGGAGGCGCTCTCGCCACCGTCGTTGGACAACCCTTCGCTCTTCGGGGTGCCTCCGACGACGA
CGGCGGTGGCGGACGGGATGGATCCGGGGGCGGATATAATGTGGGAGGAGTTGTTTTCGGGGATTCCGTTCAATGAGATAGCGGGGCCGGATACGTTCTTTTTCGATATGAACTGGGGGAGTTGACCGACCGC
GACGGTGACTGACACCTGGCGGTAGACAATCAATCCATTTCGCTATAGTTAAAGGATGGGGATGAGGGCAATTGGTTATATGATCATGTATGTAGTGGGTGTGCATAATAGTAGTGAAATGGAAGCCAAGTCAT
GTGATTGTAATCGACCGACGGAATTGAGGATATCCGGAAATACAGACACCGTGAAAGCCATGGTCTTTCCTTCGTGTAGAAGACCAGACAGACAGTCCCTGATTTACCCTGCACAAAGCACTAGAAAATTAGCAT
TCCATCCTTCTCTGCTTGCTCTGCTGATATCACTGTCATTCAATGCATAGCCATGAGCTCATCTTAGATCCAAGCACGTAATTCCATAGCCGAGGTCCACAGTGGAGCAGCAACATTCCCCATCATTGCTTTCCCCAG
GGGCCTCCCAACGACTAAATCAAGAGTATATCTCTACCGTCCAATAGATCGTCTTCGCTTCAAAATCTTTGACAATTCCAAGAGGGTCCCCATCCATCAAACCCAGTTCAATAATAGCCGAGATGCATGGTGGAGTC
AATTAGGCAGTATTGCTGGAATGTCGGGGCCAGTTCCGGGTGGTCATTGGCCGCCTGTGATG
